# Outcomes from low-risk ductal carcinoma in situ: a systematic review and meta-analysis

**DOI:** 10.1007/s10549-024-07473-w

**Published:** 2024-08-24

**Authors:** Qian Chen, Ian Campbell, Mark Elwood, Alana Cavadino, Phyu Sin Aye, Sandar Tin Tin

**Affiliations:** 1 Faculty of Medical and Health Sciences, Department of Epidemiology and Biostatistics, University of Auckland, Auckland, New Zealand; 2 Faculty of Medical and Health Sciences, Department of Surgery, University of Auckland, Auckland, New Zealand; 3 Faculty of Medical and Health Sciences, Department of Pharmacology, University of Auckland, Auckland, New Zealand; 4 Cancer Epidemiology Unit, Oxford Population Health, The University of Oxford, Oxford, UK

**Keywords:** Ductal carcinoma in situ, Low-risk DCIS, Surgery, Radiotherapy, Outcomes

## Abstract

**Purpose:**

The current standard of treatment for ductal carcinoma in situ (DCIS) is surgery with or without adjuvant radiotherapy. With a growing debate about overdiagnosis and overtreatment of low-risk DCIS, active surveillance is being explored in several ongoing trials. We conducted a systematic review and meta-analysis to evaluate the recurrence of low-risk DCIS under various treatment approaches.

**Methods:**

PubMed, Embase, Web of Science, and Cochrane were searched for studies reporting ipsilateral breast tumour event (IBTE), contralateral breast cancer (CBC), and breast cancer-specific survival (BCSS) rates at 5 and 10 years in low-risk DCIS. The primary outcome was invasive IBTE (iIBTE) defined as invasive progression in the ipsilateral breast.

**Results:**

Thirty three eligible studies were identified, involving 47,696 women with low-risk DCIS. The pooled 5-year and 10-year iIBTE rates were 3.3% (95% confidence interval [CI]: 1.3, 8.1) and 5.9% (95% CI: 3.8, 9.0), respectively. The iIBTE rates were significantly lower in patients who underwent surgery compared to those who did not, at 5 years (3.5% vs. 9.0%, P = 0.003) and 10 years (6.4% vs. 22.7%, P = 0.008). Similarly, the 10-year BCSS rate was higher in the surgery group (96.0% vs. 99.6%, P = 0.010). In patients treated with breast-conserving surgery, additional radiotherapy significantly reduced IBTE risk, but not total-CBC risk.

**Conclusion:**

This review showed a lower risk of progression and better survival in women who received surgery and additional RT for low-risk DCIS. However, our findings were primarily based on observational studies, and should be confirmed with the results from the ongoing trials.

**Supplementary Information:**

The online version contains supplementary material available at 10.1007/s10549-024-07473-w.

## Introduction

Ductal carcinoma in situ (DCIS) is characterised by the abnormal growth of epithelial cells that line breast ducts [1]. Following the implementation of breast cancer screening programs in the 1980s, there has been a significant rise in the diagnosis of DCIS. In the United States, for example, the incidence of DCIS increased dramatically from 5.8 cases per 100,000 women in the 1970s to 32.5 cases per 100,000 women in 2004; after which the rate stabilized at 68.9 per 100,000 in 2010 [2, 3].

If left untreated, DCIS may progress to invasive cancer, with the rate of approximately 30% for low and intermediate grade, and 60% for high-grade DCIS within 5–20 years of follow-up [4–6]. Most patients with DCIS are therefore treated according to guidelines, which typically involve surgery, either breast-conserving surgery (BCS) or mastectomy. They may also receive radiotherapy (RT) and endocrine therapies to reduce the risk of recurrence.

In recent years, for screen-detected low-risk DCIS, such as low histological grade, small, non-palpable lesions, there has been a growing controversy about overdiagnosis and overtreatment. Several ongoing Phase III trials are investigating the risks and benefits of active surveillance (AS) for low-risk DCIS, with the primary endpoint of ipsilateral invasive breast cancer-free survival (LORIS) [7], ipsilateral invasive breast cancer-free rate at 2 years (COMET) [8], 5 years (LORETTA) [9], or 10 years (LORD) [10]. Due to the absence of consensus on the specific definition of low-risk DCIS, these trials used slightly varying criteria based on patient age, tumour grade, estimated size and/or other pathological features. The results from these trials are not yet available. A number of observational studies have also been conducted in this field, which reported the risk of invasive cancer ranging from 0 to 25% in patients with low-risk DCIS [11–15]. Previous meta-analyses of two observational studies showed that the 10-year breast cancer-specific survival (BCSS) rates ranged from 96 to 98% with no significant difference by the receipt of surgery [16, 17].

We conducted a systematic review and meta-analysis to investigate long-term outcomes in patients with low-risk DCIS and across different treatment groups.

## Methods

This review was reported in accordance with the Preferred Reporting Items for Systematic Reviews and Meta-Analysis (PRISMA) statement [18].

### Search strategy and eligibility criteria

A systematic literature search was undertaken in Medline, Embase, Web of Science, and Cochrane library up to January 20, 2024, using the following keywords and phrases: DCIS, ductal carcinoma in situ, survival, mortality, recurrence, invasive, upstage, LORD, LORETTA, COMET, LORIS, active surveillance, low-risk, low-grade, intermediate-grade and low- intermediate grade (see details in the Supplemental Methods).

The pre-defined eligibility criteria were: (1) observational cohort studies or randomised control trials (RCTs), (2) involved women diagnosed with DCIS, with no evidence of invasion or nodal involvement, (3) reported evidence relevant to low-risk, (4) reported the outcomes of interest, (5) provided information on treatment, and (6) published in English as a full text article. Studies published only as abstracts were also included if sufficient information could be retrieved. For studies with more than one publication, the most recent or comprehensive result was included.

As the definition of low-risk DCIS definition varies across studies, we included DCIS with “low/intermediate grade” or “low-grade”, either alone or in combination with additional clinicopathologic factors (e.g. small tumour size or older age). We also considered other low-risk criteria: Oncotype DX [19] (defined low-risk as DCIS score < 39), DCISionRT test [20] (defined low-risk as decision score ≤ 2.8 without a residual risk subtype). DCIS categorized with “high-grade” or “high-risk” were excluded.

### Data extraction and quality assessment

Data extraction was performed by QC, and studies with unclear eligibility were reviewed by STT. Any discrepancies were resolved through discussion. The following information was extracted: study main author, number of eligible patients in each treatment group, year published, country, data accrual period, length of follow-up, study type, definition of low-risk DCIS, treatment types, number of patients, and outcomes.

The risk of bias was assessed by the Newcastle–Ottawa Scale (NOS) for non-randomised studies [21]. The NOS comprises eight items assessing three aspects: selection of study population, comparability of groups, and outcome for cohort studies. The NOS has a maximum score of 9, with scores higher than 7 indicating good quality, and 5–7 indicating moderate quality [21].

### Study outcomes

The primary outcome was invasive ipsilateral breast tumour event (IBTE) at 5 and 10 years, which was defined as subsequent development of invasive cancer in the ipsilateral breast.

The secondary outcomes included DCIS-IBTE, total-IBTE, and total contralateral breast cancer (CBC) at 5 and 10 years, as well as BCSS at 10 years. DCIS-IBTE was defined as recurrence/progression of DCIS in the ipsilateral breast, and total-IBTE was defined as DCIS recurrence/progression and/or invasive cancer in the ipsilateral breast. Total-CBC was defined as subsequent development of DCIS and/or invasive cancer in the contralateral breast.

### Treatment groups

The treatment groups of interest were: surgery versus no surgery; BCS versus mastectomy; BCS versus BCS followed by RT (BCS + RT); and endocrine treatment (with or without surgery and RT) versus no endocrine treatment.

### Statistical analysis

The 5-year and/or 10-year event rates estimated from the Kaplan–Meier analysis were extracted for each single-treatment study (involving only one treatment) or for each treatment group (from studies involving more than one treatment group).

Random-effects meta-analyses were undertaken, and the results were presented as the proportion of women who experienced the outcome of interest at 5 and/or 10 years (%) with 95% confidence interval (95% CI) in forest plots. The heterogeneity of results across studies was assessed using *I*^*2*^ statistic, with > 50% indicating high heterogeneity [22]. If significant heterogeneity was found, a leave-one-out sensitivity analysis was conducted to examine whether each cohort had excessive influence on the pooled analysis. For analyses involving more than 10 treatment groups, Egger’s test and funnel plots [23] were used to assess the likelihood of publication bias. If the corresponding P value from Egger’s test was less than 0.05, a funnel plot of proportion by study size was conducted to explore the potential impact from study size [24]. Subgroup analyses were conducted by treatment groups for all outcomes. In some studies, RT was optional for patients who received surgery (surgery ± RT group); this group was included in analyses comparing surgery vs. no surgery but excluded in analyses comparing BCS vs. BCS + RT. Subgroup analyses were also undertaken by study types (multi-center, single-center, and population based) and low-risk definition (grade only, and grade with other factors) for the primary outcome. All analyses were conducted using R version 4.3.2.

## Results

### Study selection

**Fig. 1:**
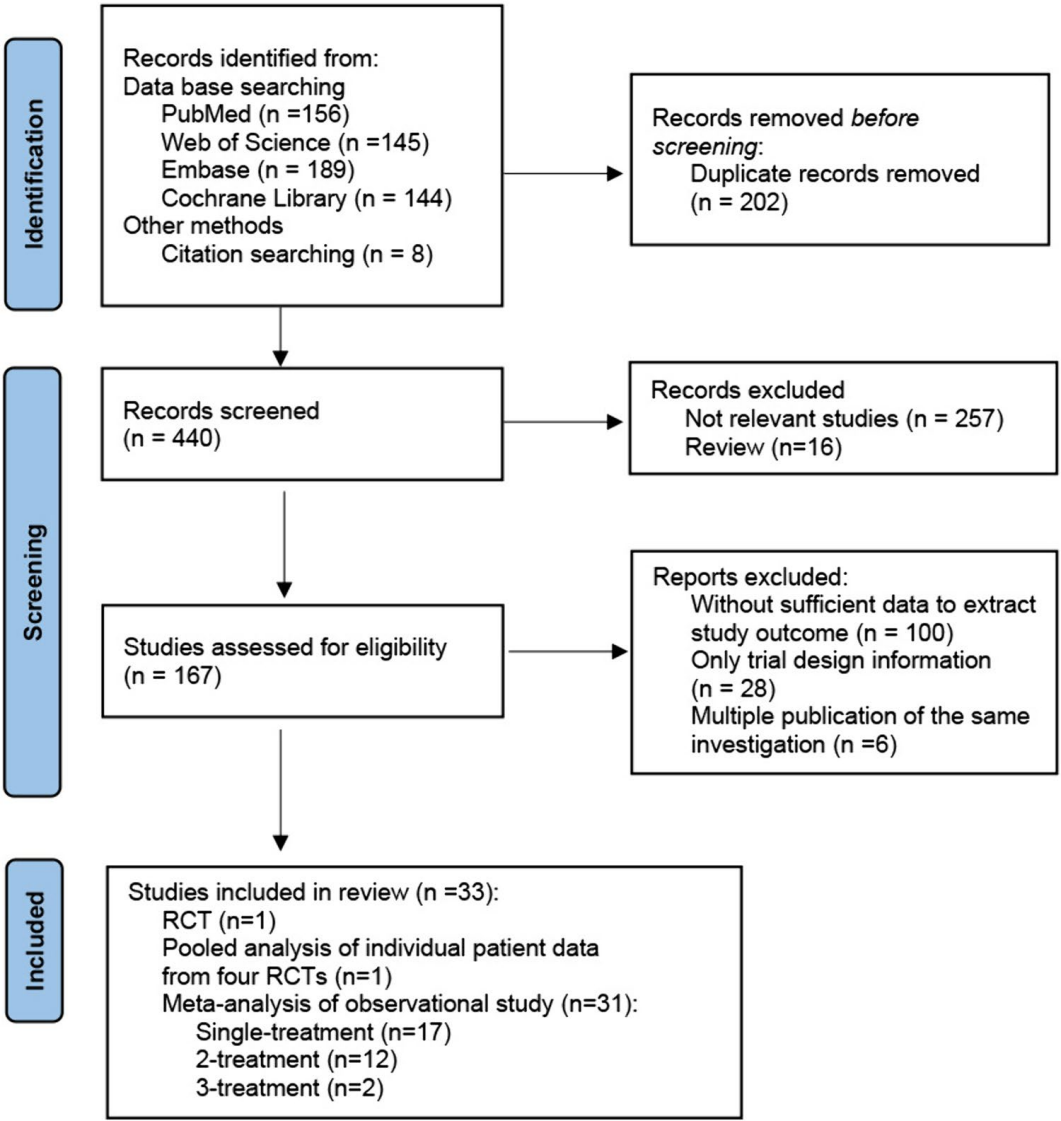
Flow diagram of search results according to PRISMA

A total of 642 related articles were retrieved (156 articles in PubMed, 145 articles in Web of Science, 189 articles in Embase, 144 articles in the Cochrane Library, and 8 articles from other sources). After removing duplicates and applying the eligibility criteria, 33 eligible studies were identified; these include: one RCT [25, 26], a pooled analysis of individual patient data from four RCTs [27], and 31 observational cohort studies [20, 28–57] published between 2000 and 2023, providing data on 47,696 women with low-risk DCIS (Fig. 1).

### Study characteristics

The Radiation Therapy Oncology Group (RTOG) 9804 [25, 26] trial, involving 629 women with low-risk DCIS, compared the effects of BCS alone vs. BCS + RT (with the optional use of tamoxifen in both groups) on 5- and 10-year recurrence rates (Table 1). An analysis by pooling individual patient data from four earlier RCTs (The Early Breast Cancer Trialists’ Collaborative Group (EBCTCG)) [27], involving 291 women with low-risk DCIS, compared BCS vs. BCS + RT for the 10-year total-IBTE.

**Table 1:** Characteristics of RCTs

Study		Country	Enrolled period	Median follow-up (years)	Sample size		Low-risk criteria	Reported outcomes
RTOG 9804 [25, 26]		US, Canada	1999–2006	13.9	629		Screen-detected DCIS < 2.5 cm, low-intermediate grade, margin greater than 3 mm	DCIS-IBTE, iIBTE, total-IBTE, total-CBC at 5, 10 years
Pooled analysis of four RCTs EBCTCG [27]	NSABP B-17	US	1985–1990	16.5	798	Low-risk subgroup 291	DCIS size 1–20 mm, low grade, negative margins	Total-IBTE at 10 years
	EORTC 10853	EU	1986–1996	10.4	918			
	SweDCIS	Sweden	1987–1999	8.4	1011			
	UK/ANZ DCIS	UK, NZ Australia	1990–1998	4.8	1002			

*CBC* contralateral breast cancer, *RCTs* randomized control trials, *RTOG 9804* Radiation Therapy Oncology Group 9804, *NSABP* National Surgical Adjuvant Breast and Bowel Project, *EORTC* European Organization for Research and Treatment of Cancer, *SweDCIS* Swedish randomized DCIS trial, *UK/ANZ* UK, Australia, and New Zealand ductal carcinoma in situ, *UK* United Kingdom, *US* United States, *IBTE* ipsilateral breast tumour event, *NZ* New Zealand

Of the 31 observational cohort studies [20, 28–57] included, 18 involved only one treatment group; 11 involved two treatment groups; and 2 involved three treatment groups (Table 2). Twenty nine studies used a retrospective [20, 28–47, 49, 50, 52–57] and two a prospective design [48, 51]. Eight studies were population-based [28, 30, 32, 33, 35, 43, 50, 57], 11 were multi-center studies [20, 34, 37, 40, 41, 45, 48, 49, 51, 55, 56], and the remaining were conducted in a single center [29, 31, 36, 38, 39, 42, 44, 46, 47, 52–54]. The results from these studies were meta-analysed, involving 46,776 women with low-risk DCIS diagnosed between 1950 and 2018. The majority of studies had a NOS score of 6–7, indicating moderate quality (see details in Supplementary Table 1). The main reason for studies not scoring higher on the NOS scale was the study design; most were retrospective, and many did not involve a comparison group.

**Table 2:** Characteristics of 31 studies included in meta-analysis of observational studies

Study	Data period	Country	Study design^a^	Study type^b^	Clinical data source	Treatment cohort^c^	Low-risk definition^d^	Mean/median follow-up (years)	Sample size	Reported outcomes^e^	NOS^f^
Vicini 2023 [20]	1986–2011	US, Australia, Sweden	R	Multi-center	Medical records	BCS; BCS + RT	DCISionRT test-Low Risk	8.5	338	DCIS-IBTE, iIBTE, total-IBTE at 10 years, total-CBC at 10 years	7
Alaeikhanehshir 2023 [28]	2005–2015	Netherlands	R	Population based (NCR)	PALGA	BCS; BCS + RT	Low-intermediate grade	8.2	4784	iIBTE at 5, 10 years	7
Zheng 2022 [29]	2010–2014	US	R	Single-center	Medical records	Surgery ± RT	COMET	NR	113	DCIS-IBTE, iIBTE, total-IBTE at 5 years	6
Maxwell 2022 [30]	2001–2018	UK	R	Population based (NHSBSP)	NCRAS	Non-surgery	Low-intermediate grade	4.1	181	iIBTE at 5, 10 years	6
You 2021 [31]	2004–2016	China	R	Single-center	Medical records	Mastectomy; BCS + RT	low-intermediate grade	NR	223	Total-IBTE at 10 years	7
Shaaban 2021 [32]	2003–2012	UK	R	Population based (NHSBSP)	NCRAS	BCS; BCS + RT	Low-intermediate grade	9.2	3152	iIBTE at 5, 10 years	7
Co 2021 [33]	1997–2006	HK	R	Population based (HKCaR)	Medical records	Non-surgery; surgery ± RT	Low-intermediate grade, age ≥ 46	11.6	372	BCSS at 10 years	7
Weinmann 2020 [34]	1990–2007	US, Sweden	R	Multi-center	Medical records	BCS; BCS + RT	DCISionRT test-Low Risk	10.4	190	DCIS-IBTE, iIBTE, total-IBTE at 10 years	7
Ryser 2019 [35]	1992–2014	US	R	Population based (SEER)	SEER	Non-surgery	Low-intermediate grade	5.5	547	iIBTE, total-CBC at 5, 10 years	6
Niwinska 2019 [36]	1996–2011	Poland	R	Single-center	Medical records	BCS; BCS + RT	RTOG 9804 criteria: DCIS < 2.5 cm, low-intermediate grade margin ≥ 3 mm	NR	133	Total-IBTE at 10 years	7
Mamtani 2019 [37]	1995–2015	US	R	Multi-center	Medical records	Mastectomy	Low-intermediate grade, age ≥ 50	NR	710	DCIS-IBTE, iIBTE, total-IBTE at 5, 10 years	6
Leonardi 2019 [38]	2000–2010	Italy	R	Single-center	Medical records	BCS + RT	Low-intermediate grade, tumour size < 2.5 cm, resection margins ≥ 3 mm	10.7	84	Total-IBTE at 5, 10 years, BCSS at 10 years	6
Martinez-Perez 2018 [39]	2000–2010	UK	R	Single-center	Medical records	BCS	Low-intermediate grade	7.4	155	DCIS-IBTE, iIBTE, total-IBTE at 5, 10 years	6
Bremer 2018 [40]	1986–2008	US, Sweden	R	Multi-center	Medical records	BCS; BCS + RT	DCISionRT test-Low Risk	9.0	196	DCIS-IBTE, iIBTE, total-IBTE at 10 years	7
Akagunduz 2018 [41]	2000–2014	Turkey	R	Multi-center	Medical records	BCS + RT	Low- intermediate grade, tumour size < 2.5 cm	4.9	98	Total-IBTE at 5 years	6
Zaremba 2017 [42]	NR	US	R	Single-center	Medical records	Mastectomy;BCS; BCS + RT	VNPI score-Low risk	NR	461	Total-IBTE at 10 years	7
Rakovitch 2017 [43]	1994–2003	Canada	R	Population based (OCR)	CIHI and medical records	BCS; BCS + RT	Oncotype DX DCIS Score-low risk	9.4	687	DCIS-IBTE, iIBTE, total-IBTE at 10 years	7
Miller 2017 [44]	1978–2011	US	R	Single-center	Medical records	BCS ± RT	Low grade	NR	536	Total-IBTE at 10 years, total -CBC at 5,10 years	6
Khan 2017 [45]	NR	US	R	Multi-center	Medical records	BCS	Low-intermediate grade	6.5	475	DCIS-IBTE, iIBTE, total-IBTE at 5,10 years, BCSS at 10 years	6
Pilewskie 2016 [46]	1996–2011	US	R	Single-center	Medical records	BCS; BCS + RT	LORIS	5.9	400	DCIS-IBTE, iIBTE, total-IBTE at 5,10 years	7
Frank 2016 [47]	1983–2013	France	R	Single-center	Medical records	Mastectomy; BCS; BCS + RT	Low grade	6.7	329	Total-IBTE at 5, 10 years	7
Solin 2015 [48]	1997–2002	US	P	Multi-center	Medical records	BCS	Low- intermediate grade, tumour size < 2.5 cm	12.3	561	DCIS-IBTE, iIBTE, total-IBTE, total-CBC at 5,10 years (Biopsy)	6
Sanders 2015 [49]	1950–1989	US	R	Multi-center	Medical records	Non-surgery	Low grade	10.0	28	iIBTE at 5, 10 years	6
Sagara 2015 [50]	1998–2014	US	R	Population based (SEER)	SEER	Non-surgery; surgery	Low- intermediate grade	6.0	31,106	BCSS at 10 years	8
Wong 2014 [51]	1995–2002	US	P	Multi-center	Medical records	BCS	Low- intermediate grade, tumour size < 2.5 cm, margin ≥ 1 cm	11.0	143	DCIS-IBTE, iIBTE, total-IBTE, total-CBC at 5, 10 years (Mamography)	6
Wong 2014 [52]	1995–2011	Singapore	R	Single-center	Medical records	BCS + RT	Low-intermediate grade	5.0	219	Total-IBTE at 5, 10 years	6
Kim 2014 [53]	1999–2009	Korea	R	Single-centre	Medical records	BCS	Low- intermediate grade, tumour size < 2.5 cm, margin ≥ 0.3 cm	4.8	99	Total-IBTE at 5 years	6
Goyal 2011 [54]	2002–2004	US	R	Single-center	Medical records	BCS + RT	Low- intermediate grade, tumour size < 2.5 cm	4.4	41	DCIS-IBTE, iIBTE, Total-IBTE, total-CBC at 5 years	6
Motwani 2010 [55]	1980–2009	US	R	Multi-center	Medical records	BCS + RT	Low- intermediate grade, tumour size < 2.5 cm	6.9	196	Total-IBTE, total-CBC at 5 years	6
MacAusland 2007 [56]	1987–2004	US	R	Multi-center	Medical records	BCS	VNPI score-low risk	4.6	159	Total-IBTE at 5 years	6
Ringberg 2000 [57]	1987–1991	Sweden	R	Population based (Regional Tumour Registry in Lund)	Medical records	BCS	Low-intermediate grade	5.3	60	Total-IBTE at 5 years	6

*BCS* breast-conserving surgery, *CBC* contralateral breast cancer, *CIHI* Canadian Institute for Health Information, *COMET* Comparison of Operative versus Monitoring and Endocrine Therapy, *HK* Hong Kong, *HKCaR* Hong Kong Cancer Registry, *LORIS* Low Risk Ductal Carcinoma In Situ, *LORD* LOw Risk DCIS, *NR* not reported, *IBTE* ipsilateral breast tumour event, *OCR* Ontario Cancer Registry, *PALGA* The Nationwide Network and Registry of Histo- and Cytopathology in the Netherlands, *P* Prospective cohort, *R* Retrospective cohort, *RT* Radiotherapy, *SEER* Surveillance, Epidemiology, and End Results Program, *VNPI* Van Nuys Prognostic Index, *NCR* Netherlands Cancer Registry, *NCRAS* National Cancer Registration and Analysis Service, *NHSBSP* UK National Health Service Breast Screening Programme, *NOS* Newcastle–Ottawa Scale, *UK* United Kingdom, *US* United States

^a^Study design: P, prospective study; R, retrospective study

^b^Study type: For population based study, the agency of registry was specified

^c^Treatment: If the study specifies the type of surgery, the specific surgery is recorded; otherwise, it is recorded as surgery

^d^Key low-risk definitions from prognostic tests or clinical trials: DCISionRT test-Low Risk: decision score <  = 2.8 without a residual risk subtype; VNPI score-low risk: VNPI score 4–6; Oncotype DX DCIS Score-low risk: DCIS score < 39; COMET: aged 40 or older, low-intermediate grade, hormone receptor-positive, DCIS diagnosed by biopsy, absence of nipple discharge; LORIS: aged 46 or older, screen-detected calcifications, low-intermediate grade DCIS diagnosed by biopsy, absence of nipple discharge

^e^Outcome: For retrospective studies, outcomes were retrieved from registry or medical records, while for prospective studies, the assessments of outcomes were specified

^f^The overall risk of bias was based on NOS score. NOS has a maximum score of 9, with scores higher than 7 indicating good quality, and 5–7 indicating moderate quality

### Results of RCTs

The RTOG 9804 trial reported significantly lower IBTE rates in women who received BCS + RT in comparison to those who received BCS alone at 5 years (total-IBTE: 0.4% vs. 3.5%, P < 0.001) and 10 years (iIBTE: 0.4% vs. 4.3%, P < 0.001; total-IBTE: 1.5% vs. 9.2%, P < 0.001) [25, 26]. Similarly, in the EBCTCG pooled analysis, the 10-year total-IBTE was lower in women who received BCS + RT compared to those who received BCS (12.1% vs. 30.1%, P = 0.002) [27]. The total-CBC was similar between the two treatment groups in the RTOG 9804 trial (5-year: 3.4% vs. 2.2%, P = 0.86; 10-year: 5.5% vs. 4.6%, P = 0.27) [25, 26]. Neither study has assessed the outcomes of interest in relation to endocrine treatment.

### Meta-analysis of observational studies

#### Pooled 5- and 10-year event and survival rates

Thirteen studies (ten single-treatment [29, 30, 35, 37, 39, 45, 48, 49, 51, 54], three 2-treatment [28, 32, 46]) and 15 studies (eight single-treatment [30, 35, 37, 39, 45, 48, 49, 51], seven 2-treatment [20, 28, 32, 34, 40, 43, 46]) reported iIBTE rates at 5 and 10 years respectively. The estimated pooled iIBTE rates were 3.3% (95% CI, 1.3–8.1) at 5 years and 5.9% (95% CI, 3.8–9.0) at 10 years (Table 3; Supplementary Figures 1, 2).

**Table 3:** Pooled 5- and 10-year event and survival rates

Variable	5-year event				10-year event				
	No.of Tx groups	No. of patients	5-year rate (95% CI)	I^2^, %		No.of Tx groups	No. of patients	10-year rate (95% CI)	I^2^, %
iIBTE	16	11,290	3.3 (1.3,8.1)	94.53		22	12,547	5.9 (3.8,9.0)	92.90
DCIS-IBTE	9	2598	3.9 (2.3,6.3)	56.95		15	3855	5.0 (3.4,7.4)	68.83
Total-IBTE	19	3842	5.7 (3.8,8.3)	68.48		28	5840	8.7 (6.6,11.3)	79.40
Total-CBC	6	2024	3.2 (2.0,5.1)	41.00		5	1911	5.6 (4.3,7.4)	7.95
BCSS	/	/	/	/		6	32,037	98.8 (95.3, 99.7)	87.03

*BCSS* breast cancer specific survival, *iIBTE* invasive ipsilateral breast tumour event, *DCIS-IBTE* DCIS ipsilateral breast tumor event, *Total-IBTE* DCIS and/or invasive ipsilateral breast tumour event, *Total-CBC* DCIS and/or invasive contralateral cancer, *Tx* treatment

Eight studies (seven single-treatment [29, 37, 39, 45, 48, 51, 54], one 2-treatment [46]) reported DCIS-IBTE at 5 years, and 10 studies (five single-treatment [37, 39, 45, 48, 51], five 2-treatment [20, 34, 40, 43, 46]) at 10 years. The estimated pooled rates were 3.9% (95% CI, 2.3–6.3) at 5 years and 5.0% (95% CI, 3.4–7.4) at 10 years (Table 3; Supplementary Figures 3, 4).

Sixteen studies (fourteen single-treatment [29, 37–39, 41, 45, 48, 51–57], one 2-treatment [46], one 3-treatment [47]) reported total-IBTE rates at 5 years and 17 studies (eight single-treatment [37–39, 44, 45, 48, 51, 52], seven 2-treatment [20, 31, 34, 36, 40, 43, 46], two 3-treatment [42, 47]) at 10 years, with the pooled rates of 5.7% (95% CI, 3.8–8.3) and 8.7% (95% CI, 6.6–11.3), respectively (Table 3; Supplementary Figures 5, 6).

Six studies (six single-treatment [35, 44, 48, 51, 54, 55]) reported total-CBC rates at 5 years with a pooled rate of 3.2% (95% CI, 2.0–5.1), and five studies (four single-treatment [35, 44, 48, 51], one 2-treatment [20]) at 10 years with a pooled rate of 5.6% (95% CI, 4.3–7.4). Furthermore, four studies (two single-treatment [38, 45] and two 2-treatment [33, 50]) reported a pooled 10 -year BCSS rate of 98.8% (95% CI, 95.3–99.7) in low-risk DCIS (Table 3; Supplementary Figures 7, 8, 9).

### Subgroup analyses by treatment groups

#### Surgery vs. no surgery

**Fig. 2:**
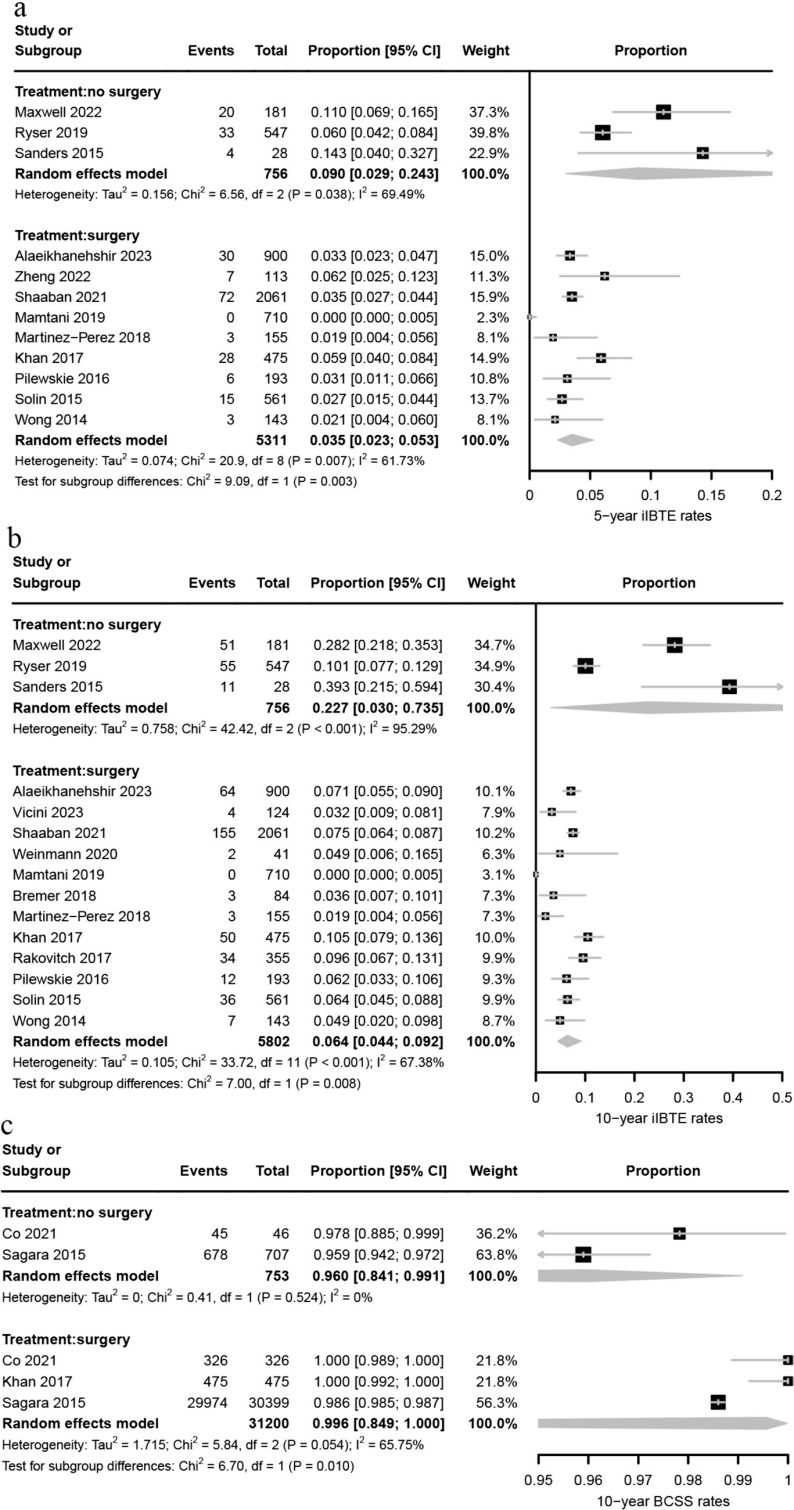
Pooled analysis of 5-year **a**, 10-year **b** iIBTE rates and 10-year BCSS rates **c** in low-risk DCIS comparing no surgery and surgery

The iIBTE rates were significantly lower in patients who underwent surgery compared to those who did not, at 5 years (3.5% vs. 9.0%, P = 0.003; Fig. 2a) and 10 years (6.4% vs. 22.7%, P = 0.008; Fig. 2b). Similarly, the 10-year BCSS rate was higher in the surgery group (96.0% vs. 99.6% P = 0.010) (Fig. 2c). No study reported DCIS-IBTE, total-IBTE and total-CBC in no surgery group.

#### Mastectomy vs. BCS

In women who received surgery alone, those who underwent mastectomy had a significantly lower rate of total-IBTE compared to those who underwent BCS (0.6% vs. 7.5%, P = 0.161; 10-year, 1.4% vs. 12.7%, P = 0.011) (Supplementary Figures 10, 11). Only one study [37] reported iIBTE and DCIS-IBTE in patients who underwent mastectomy with a 0% recurrence rate. No study compared CBC and BCSS in the two groups or assessed the effect of margin status on the outcomes of interest.

#### BCS vs. BCS + RT

The iIBTE, DCIS-IBTE, and total-IBTE rates were lower in patients who received RT at 5 years (iIBTE: 1.3% vs. 3.5%, P < 0.001; DCIS-IBTE: 1.8% vs. 4.2%, P < 0.001; total-IBTE: 3.4% vs. 7.5%, P = 0.026) and 10 years (iIBTE: 3.9% vs. 6.9%, P = 0.004; DCIS-IBTE: 3.1% vs. 7.2%, P < 0.001; total-IBTE: 6.7% vs. 12.7%, P < 0.001) (Supplementary Figure 12). Similarly, the pooled total-CBC rate at 5 years was lower in the BCS + RT group although not significant (2.4% vs. 4.5%, P = 0.074) (Supplementary Figure 13). No study reported a 10-year total-CBC rate in patients who underwent RT following BCS, but one study [38] reported a 100% 10-year BCSS rate in this treatment group.

#### Endocrine treatment vs. no endocrine treatment

No study assessed the outcomes of interest in relation to endocrine treatment.

### Other subgroup analyses

There were no significant differences in the pooled iIBTE rates by study type or by the definition of low-risk (Table 4; Supplementary Figures 14, 15, 16, 17).

**Table 4:** The 5-year and 10-year iIBTE rates subgroup analysis by study type and low-risk definition

Variable	5-year iIBTE					10-year iIBTE				
	No.of Tx groups	No. of patients	5-year rate (95% CI)	I^2^, %	P value	No.of Tx groups	No. of patients	10-year rate (95% CI)	I^2^, %	P value
Study type subgroups										
Single-center	5	709	2.6 (0.9,7.7)	50.53	0.897	3	555	4.7 (1.4,14.8)	41.62	0.284
Multi-center	5	1917	2.9 (0.4,20.6)	82.23		11	2641	4.6 (1.9,10.7)	85.51	
Population based	6	8664	3.3 (1.3,8.1)	94.53		8	9351	7.9 (4.4,13.9)	96.81	
Low-risk characteristics subgroups										
Grade only	9	9322	3.9 (2.0,7.5)	92.79	0.237	9	9322	8.8 (4.0,18.2)	96.94	0.123
Grade and other factors	7	1968	2.1 (0.7,6.0)	60.75		13	3225	4.9 (3.3,7.1)	62.06	

*iIBTE* invasive ipsilateral breast tumour event, *Tx* treatment

### Publication bias and sensitivity analysis

Egger’s test and funnel plots showed no publication bias for 5- and 10-year iIBTE rates. However, there was evidence of publication bias for the 10-year DCIS-IBTE rate, 5- and 10-year total IBTE rates (P < 0.05) (Supplementary Figures 18, 19, 20, 21, 22). The funnel plot of proportion by sample size revealed that the publication bias of these outcomes appeared to be driven by higher proportion of the outcome of interest in smaller studies (Supplementary Figures 23, 24, 25).

The sensitivity analysis revealed that removing one study at a time from the pooled analysis did not substantially alter the result, indicating that results were reliable (Supplementary Figures 26, 27, 28, 29, 30, 31, 32).

## Discussion

In this meta-analysis, the pooled iIBTE rate in women with low-risk DCIS was 3.3% at 5 years and 5.9% at 10 years. Compared to patients who did not receive surgery, those who underwent surgery for low-risk DCIS had lower iIBTE rates, and there was also a trend towards improved 10-year BCSS rates. In comparison to BCS, mastectomy and the additional RT after BCS were also associated with a reduced rate of IBTE.

Several population-based studies have investigated long-term progression risk and survival outcomes in women with DCIS in general. In the Netherlands, the iIBTE risks at 15 years for screen-detected and non-screen-detected DCIS diagnosed between 1989 and 2004 were reported as 6% and 7%, respectively [58]. In England, Mannu et al. found the 5- and 15-year invasive breast cancer risks of 3.7% and 5.1%, and 12.3% and 15.4%, respectively, for screen-detected and non-screen-detected DCIS diagnosed between 2000 and 2009 [59]. Using the data from surveillance, epidemiology, and end results (SEER) from 1988 to 2011, Narod et al. [60] reported that the 20-year iIBTE risk and invasive CBC of DCIS treated with surgery, with or without RT, was 5.9% and 6.2%. A review of studies published between 2000 and 2015 reported that the annual risk of contralateral breast cancer ranged from 0.5 to 0.75% [61]. As expected, the ipsilateral and contralateral progression risk observed in our review for low-risk DCIS is lower than that reported in previous studies on DCIS in general.

As for DCIS in general, current treatment options for low-risk DCIS involve surgery, often followed by RT and endocrine treatment. However, there has been a debate about overtreatment; for example, there was a preference for AS over conventional treatment in an Australian study on women’s preference [62] and the LORD trial [63], but health professionals from the United State [64], Australia and New Zealand [65] expressed reservation about recommending AS for low-risk DCIS. Our pooled analysis showed that patients who received surgery had lower invasive breast cancer rates, which may contribute to better survival compared to those under AS. The addition of RT after BCS was also associated with a reduced rate of IBTE, but had no influence on CBC in observational analyses as well as in the RCTs [25, 26]. AS is not yet standard care for DCIS and has only been offered in the research settings. Therefore, patients who opted for AS in the observational studies included in this meta-analysis may either have comorbidities, making them unfit for surgery, or have very small tumour sizes with favourable pathologic features, which could potentially bias survival rate comparisons. Previous analysis of SEER data showed higher overall and breast cancer specific mortality in the AS group compared to the treatment group among older DCIS patients [66].

While we were not able to assess the use and effect of endocrine treatment for oestrogen receptor (ER) positive low risk DCIS, the NSABP B-24 trial showed that tamoxifen significantly reduced ipsilateral and contralateral event in those with ER-positive DCIS in general [67]. The NSABP B-35 trial [68] also revealed that Anastrozole significantly reduced CBC compared to tamoxifen in patients with ER-positive DCIS, who underwent BCS and RT. An ongoing study is investigating whether low‑dose tamoxifen is non‑inferior to RT following BCS in preventing IBTE in low-risk DCIS [69]. Endocrine therapy is also an option in some of the current AS trials [8, 9].

In defining low-risk DCIS, tumour grade, size and margin may be considered. Ongoing phase III trials (COMET [8], LORIS [7], LORD [10]), comparing surgery with AS, share similar definitions of low-risk, typically involving patients aged 40 years or older, and non-high-grade DCIS, and limited size. Although human epidermal growth factor receptor 2 (HER2) expression is not routinely measured for DCIS as in invasive breast cancer, HER2-positive DCIS has been associated with a higher proportion of high-grade tumour and an increased risk of DCIS-IBTE [70]. The COMET trial [8] further requires HER2-negative DCIS in the eligibility.

However, the absence of consensus on the definition of low-risk DCIS, together with varying frequencies and methods of follow-up in observational studies, presents challenges for meta-analysing the results. Non-high grade is considered one of the favourable prognostic factors associated with lower IBTE rates after surgery [71–73]. Tumour size did not emerge as a significant prognostic factor in the NSABP B-17 and B-24 studies [72] which primarily involved relatively small DCIS (≤ 2 cm), probably due to poor estimates of size in these studies. Regarding margins, a previous meta-analysis [74] confirmed a higher 10-year local recurrence in DCIS with < 2 mm negative margin in patients who received BCS alone, but the rate did not differ by additional RT. Schmitz et al. conducted pooled analysis of four cohort studies and found that larger tumour size and positive margins were associated with an increased risk of recurrence in DCIS [75]. We were not able to stratify the results by margin status (due to limited research undertaken to date for low-risk DCIS) but our subgroup analysis indicated that the definition of low-risk, which took into account grade alone or in combination with other factors, did not significantly affect the pooled iIBTE rates.

To our knowledge, this review is the first systematic analysis to summarise and pool 5- and 10-year breast event and survival rates in low-risk DCIS across different treatment options. We, however, included only studies published in English, with the majority from Europe and North America, which may limit the generalizability of the findings. A potential bias may be present due to smaller studies reporting higher 5- or 10-year outcomes compared to larger studies, suggesting that smaller studies with lower rates were less likely to report these outcomes and were therefore excluded from these analyses. We were not able to examine the outcome of low-risk DCIS across different age groups. This is an area for future research given the potential impact of age on DCIS post-surgery recurrence, with a lower risk in older women [76].

In summary, this review showed a lower risk of progression and better survival in women who received surgery and additional RT for low-risk DCIS. However, our results were mostly observational and should be confirmed with those from the ongoing trials.

## Supplementary Information

Below is the link to the electronic supplementary material.

Supplementary file1 (DOCX 10489 KB)

## Data Availability

No datasets were generated or analysed during the current study.
