# Supplementary material for: Outcomes from low-risk ductal carcinoma in situ: a systematic review and meta-analysis
Source: Breast Cancer Res Treat. 2024 Aug 24;208(2):237–51. doi: 10.1007/s10549-024-07473-w (PMC11457553; doi:10.1007/s10549-024-07473-w)
Supplement: Supplementary file 1 — Supplementary file1 (DOCX 10489 KB) [file 10549_2024_7473_MOESM1_ESM.docx]

**Supplemental Materials**

Supplemental method- Searching strategies

| **Database** | **Search String** | **Results** |
| --- | --- | --- |
| MEDLINE (PubMed) | (((((((DCIS[Title/Abstract]) OR (ductal carcinoma in situ[Title/Abstract])) AND ((((((((((low risk) OR (low grade)) OR (low-intermediate grade)) OR (good risk)) OR (intermediate grade)) OR (LORD)) OR (LORETTA)) OR (COMET)) OR (LORIS)) OR (active surveillance))) AND (((((ipsilateral) OR (contralateral)) OR (survival)) OR (disease-specific survival)) OR (breast cancer specific survival))) AND ((((5 year) OR (10 year)) OR (five years)) OR (ten years))) AND ((((((((survival[Title/Abstract]) OR (mortality[Title/Abstract])) OR (recurrence[Title/Abstract])) OR (invasive[Title/Abstract])) OR (upstage[Title/Abstract])) OR (outcome[Title/Abstract])) OR (relapse[Title/Abstract])) OR (disease progression[Title/Abstract]))) AND (English[Language])) NOT (((Case Reports[Publication Type]) OR (Review[Publication Type])) OR (Comment[Publication Type])) | 156 |
| Embase | (((DCIS or ductal carcinoma in situ) and (survival or mortality or recurrence or Relapse or disease progression or invasive or upstage or outcome)).ab. and (LORD or LORETTA or COMET or LORIS or active surveillance or low-risk or low-grade or low-intermediate grade or good risk or intermediate grade).af. and (ipsilateral or contralateral or survival or disease-specific survival or breast cancer specific survival).af. and (5 year or 10 year or five years or ten years).af. and English.lg.) not (case reports or comment or review).pt. | 145 |
| Web of Science | DCIS or ductal carcinoma in situ (Abstract) AND survival or mortality or recurrence or Relapse or disease progression or invasive or upstage or outcome (Abstract) AND LORD or lorette or COMET or LORIS or active surveillance or low-risk or low-grade or low-intermediate grade or good risk or intermediate grade (All Fields) AND ipsilateral or contralateral or survival or disease-specific survival or breast cancer specific survival (All Fields) AND 5 year or 10 year or five years or ten years (All Fields) AND English (Language) NOT Review (Document Type) NOT Withdrawn Publication (Document Type) NOT Letter (Document Type) | 189 |
| Cochrane library | DCIS or ductal carcinoma in situ in Title Abstract Keyword AND survival or mortality or recurrence or Relapse or disease progression or invasive or upstage or outcome or ipsilateral or contralateral in All Text AND LORD or LORETTA or COMET or LORIS or active surveillance or low-risk or low-grade or low-intermediate grade or good risk or intermediate grade in All Text AND English in Language NOT case reports or comment or review or letters in Publication Type | 144 |
| Citation searching |  | 8 |

Supplementary table

Supplementary Table 1. Quality Assessment

| **Study** | **Representative-ness of exposed cohort** | **Selection of non-exposed cohort** | **Ascertain-ment of exposure** | **Demonstration that outcome of interest was not present at start of study** | **Comparability of cohorts on the basis of design or analysis** | **Assessment of outcome** | **Was follow-up long enough for outcomes to occur?** | **Adequacy of follow-up of cohorts** | **Total score** |
| --- | --- | --- | --- | --- | --- | --- | --- | --- | --- |
| Vicini 2023 | 1 | 1 | 1 | 1 | 0 | 1 | 1 | 1 | 7 |
| Alaeikhanehshir 2023 | 1 | 1 | 1 | 1 | 0 | 1 | 1 | 1 | 7 |
| Zheng 2022 | 1 | 0 | 1 | 1 | 0 | 1 | 1 | 1 | 6 |
| Maxwell 2022 | 1 | 0 | 1 | 1 | 0 | 1 | 1 | 1 | 6 |
| You 2021 | 1 | 1 | 1 | 1 | 0 | 1 | 1 | 1 | 7 |
| Co 2021 | 1 | 1 | 1 | 1 | 0 | 1 | 1 | 1 | 7 |
| Shaaban 2020 | 1 | 1 | 1 | 1 | 0 | 1 | 1 | 1 | 7 |
| Weinmann2020 | 1 | 1 | 1 | 1 | 0 | 1 | 1 | 1 | 7 |
| Niwinska 2019 | 1 | 1 | 1 | 1 | 0 | 1 | 1 | 1 | 7 |
| Mamtani 2019 | 1 | 0 | 1 | 1 | 0 | 1 | 1 | 1 | 6 |
| Ryser2019 | 1 | 0 | 1 | 1 | 0 | 1 | 1 | 1 | 6 |
| Leonardi 2019 | 1 | 0 | 1 | 1 | 0 | 1 | 1 | 1 | 6 |
| Akagunduz2018 | 1 | 0 | 1 | 1 | 0 | 1 | 1 | 1 | 6 |
| Bremer2018 | 1 | 1 | 1 | 1 | 0 | 1 | 1 | 1 | 7 |
| Martinez-Perez2018 | 1 | 0 | 1 | 1 | 0 | 1 | 1 | 1 | 6 |
| Zaremba 2017 | 1 | 1 | 1 | 1 | 0 | 1 | 1 | 1 | 7 |
| Khan2017 | 1 | 0 | 1 | 1 | 0 | 1 | 1 | 1 | 6 |
| Miller 2017 | 1 | 0 | 1 | 1 | 0 | 1 | 1 | 1 | 6 |
| Rakovitch2016 | 1 | 1 | 1 | 1 | 0 | 1 | 1 | 1 | 7 |
| Frank 2016 | 1 | 1 | 1 | 1 | 0 | 1 | 1 | 1 | 7 |
| Pilewskie2016 | 1 | 1 | 1 | 1 | 0 | 1 | 1 | 1 | 7 |
| Sagara 2015 | 1 | 1 | 1 | 1 | 1 | 1 | 1 | 1 | 8 |
| Solin2015 | 1 | 0 | 1 | 1 | 0 | 1 | 1 | 1 | 6 |
| Sanders 2015 | 1 | 0 | 1 | 1 | 0 | 1 | 1 | 1 | 6 |
| Wong2014 | 1 | 0 | 1 | 1 | 0 | 1 | 1 | 1 | 6 |
| Wong2014 | 1 | 0 | 1 | 1 | 0 | 1 | 1 | 1 | 6 |
| Kim 2014 | 1 | 0 | 1 | 1 | 0 | 1 | 1 | 1 | 6 |
| Goyal2011 | 1 | 0 | 1 | 1 | 0 | 1 | 1 | 1 | 6 |
| Motwani2010 | 1 | 0 | 1 | 1 | 0 | 1 | 1 | 1 | 6 |
| MacAusland2007 | 1 | 0 | 1 | 1 | 0 | 1 | 1 | 1 | 6 |
| Ringberg2000 | 1 | 0 | 1 | 1 | 0 | 1 | 1 | 1 | 6 |

Supplementary Figures


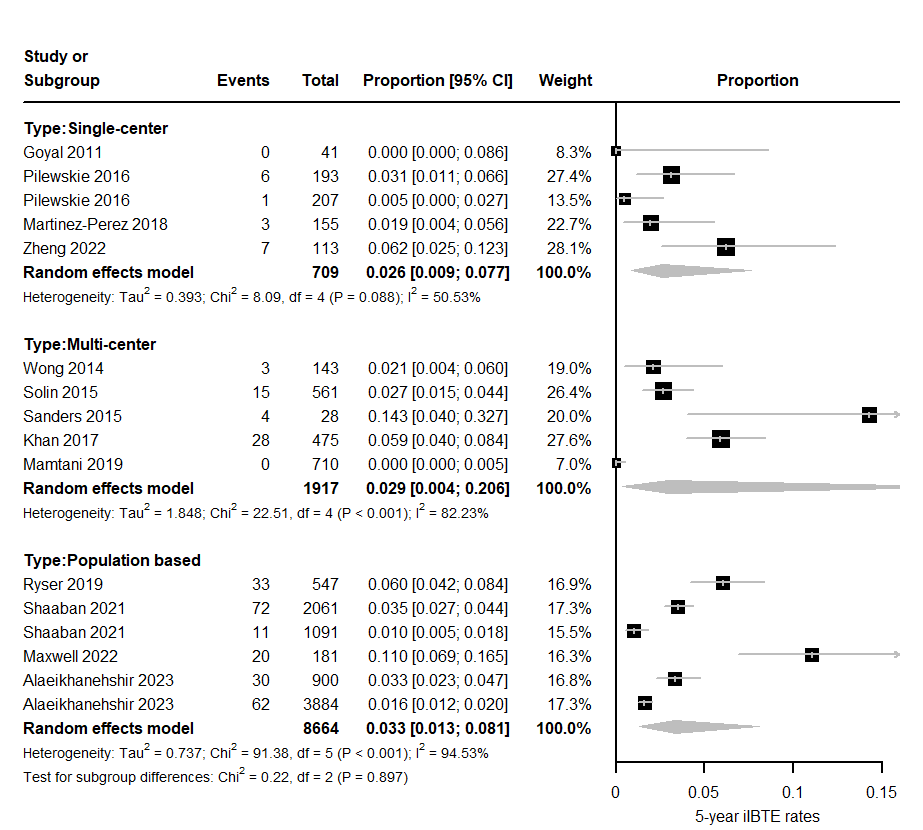


Supplementary Figure 1. Pooled analysis of 5-year iIBTE rates by treatments


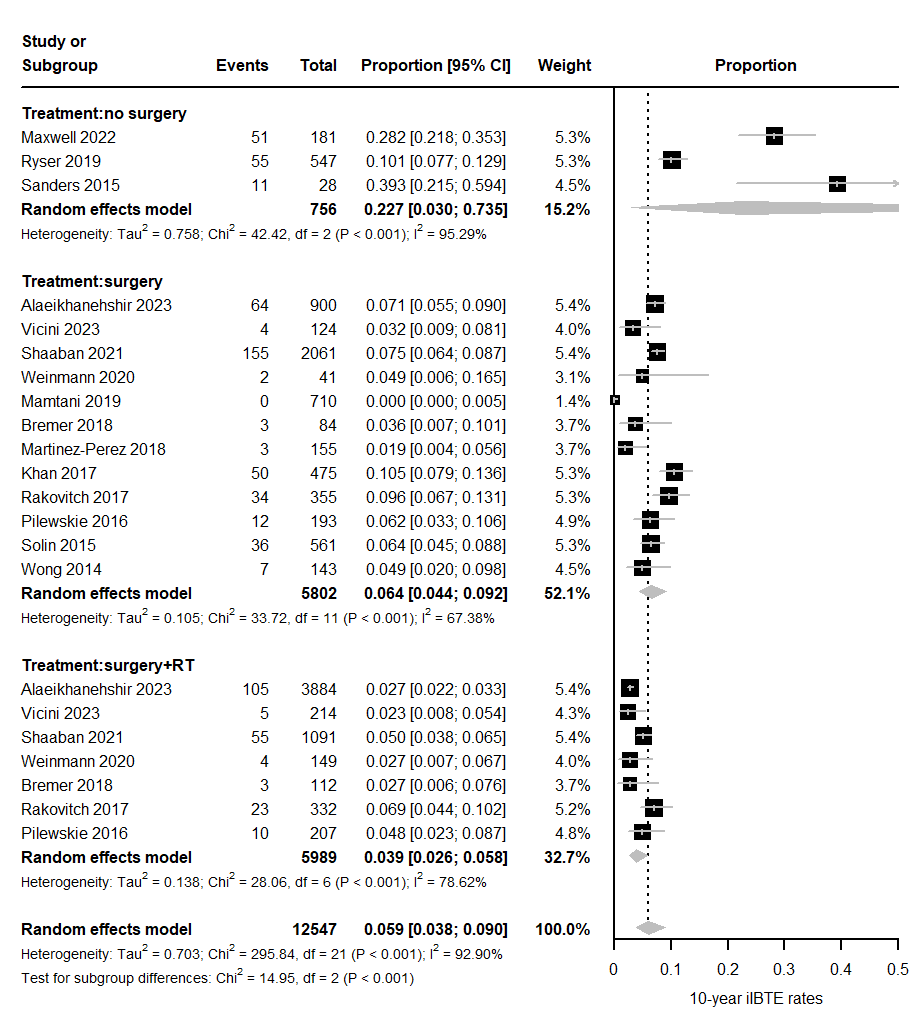


Supplementary Figure 2. Pooled analysis of 10-year iIBTE rates by treatments


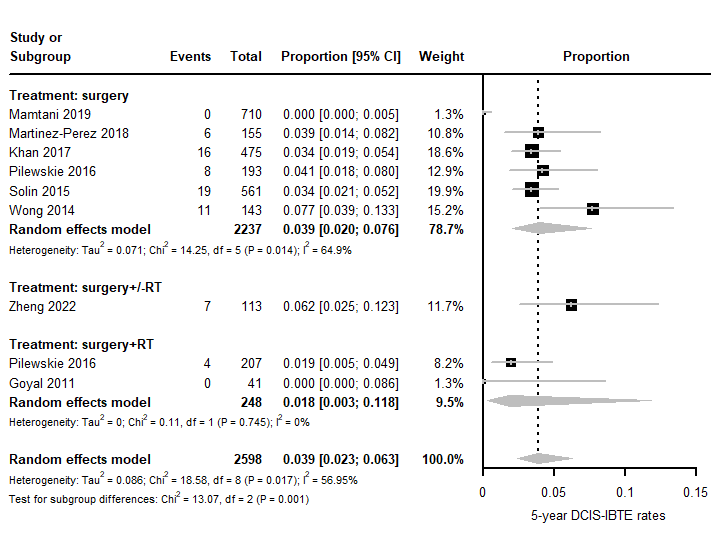


Supplementary Figure 3. Pooled analysis of 5-year DCIS-IBTE rates by treatments


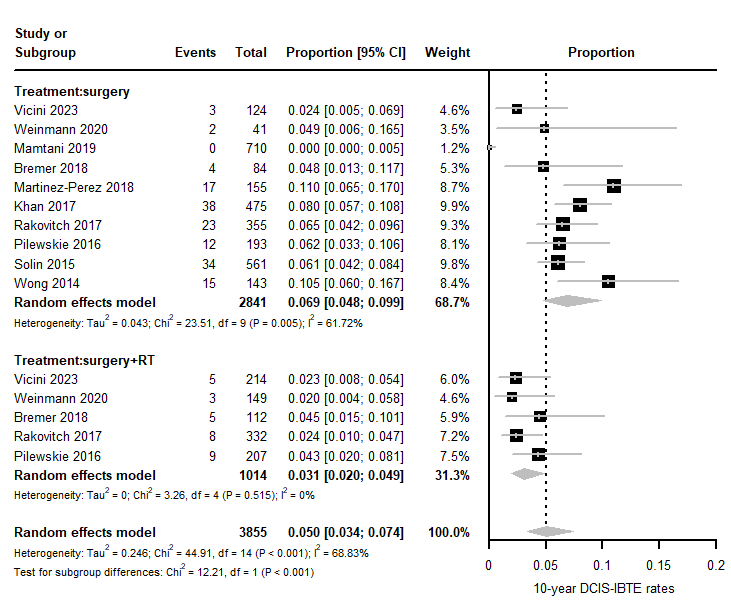


Supplementary Figure 4. Pooled analysis of 10-year DCIS-IBTE rates by treatments


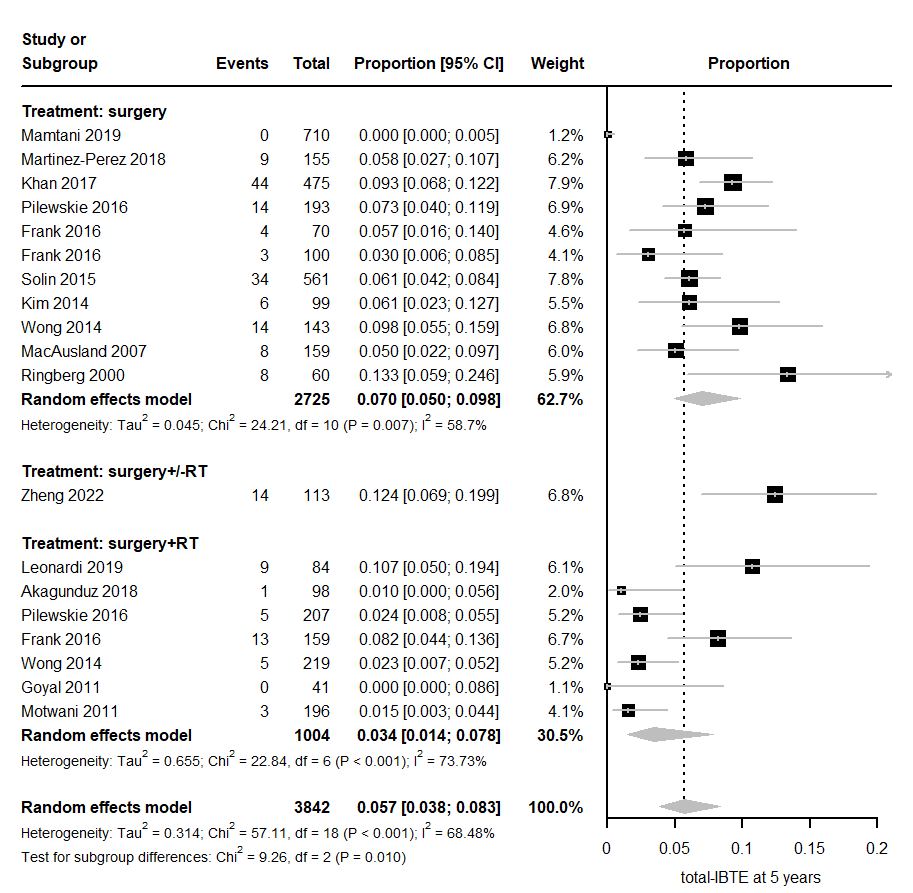


Supplementary Figure 5. Pooled analysis of 5-year total-IBTE rates by treatments


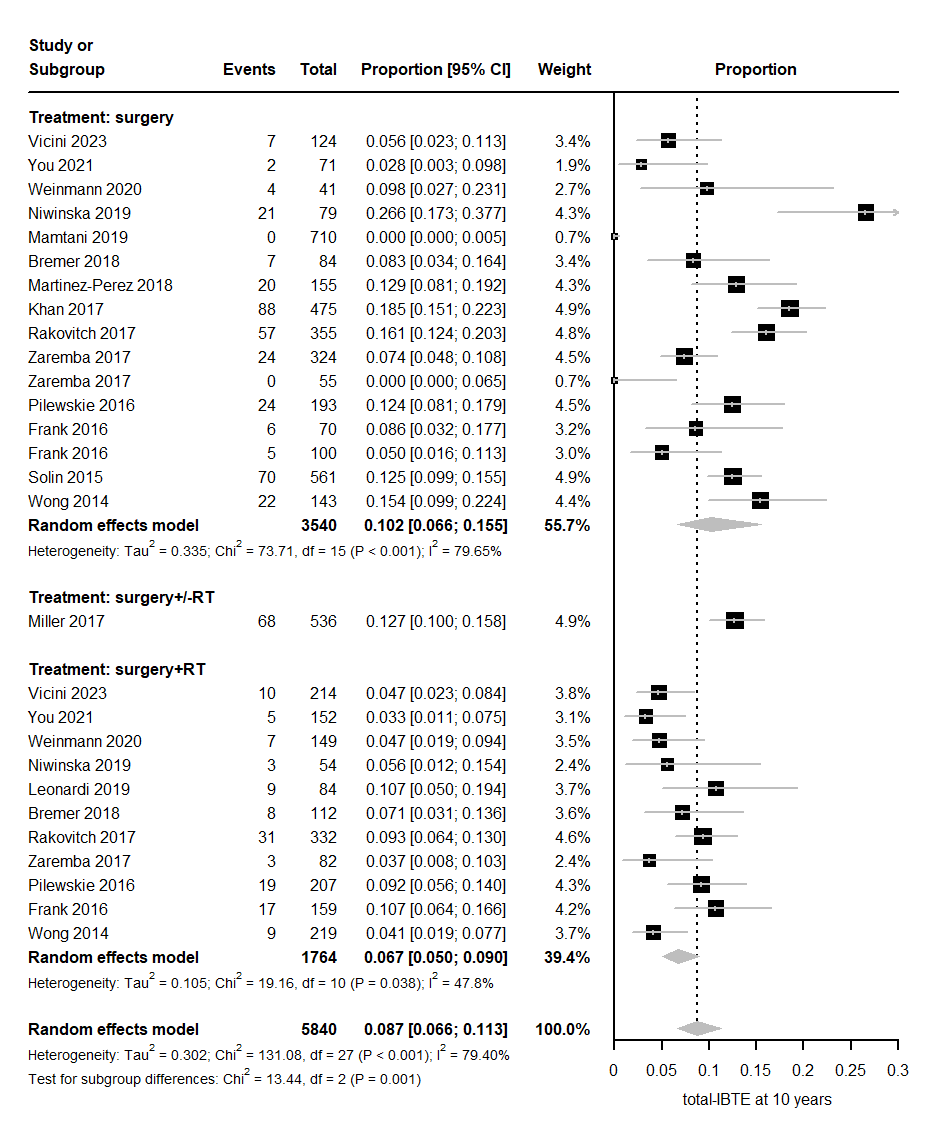


Supplementary Figure 6. Pooled analysis of 10-year total-IBTE rates by treatments


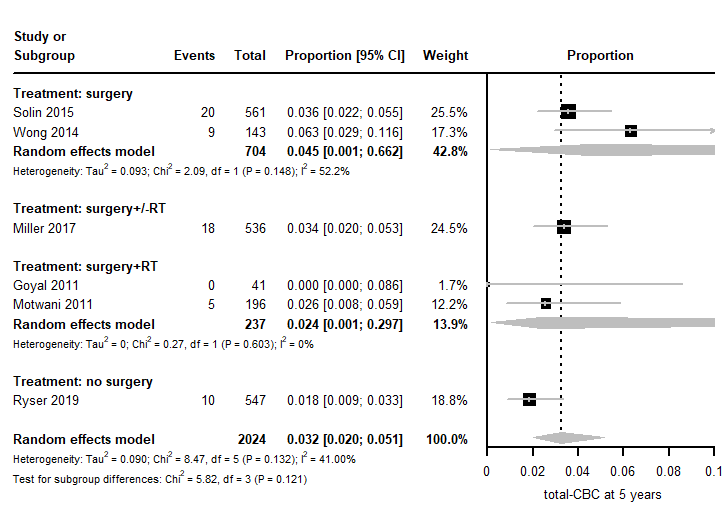


Supplementary Figure 7. Pooled analysis of 5-year total-CBC rates by treatments





Supplementary Figure 8. Pooled analysis of 10-year total-CBC rates by treatments


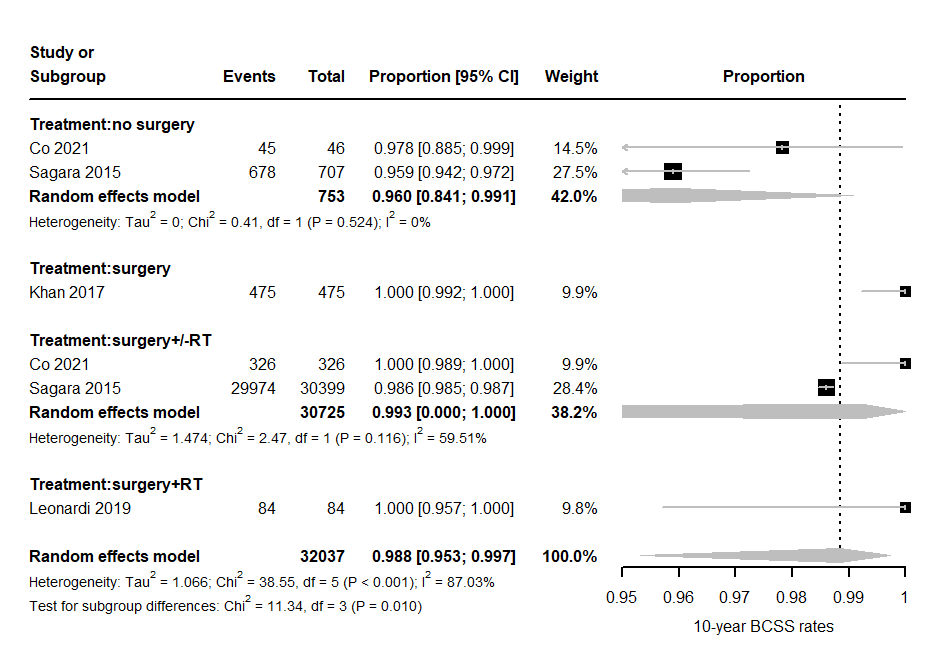


Supplementary Figure 9. Pooled analysis of 10-year BCSS rates by treatments


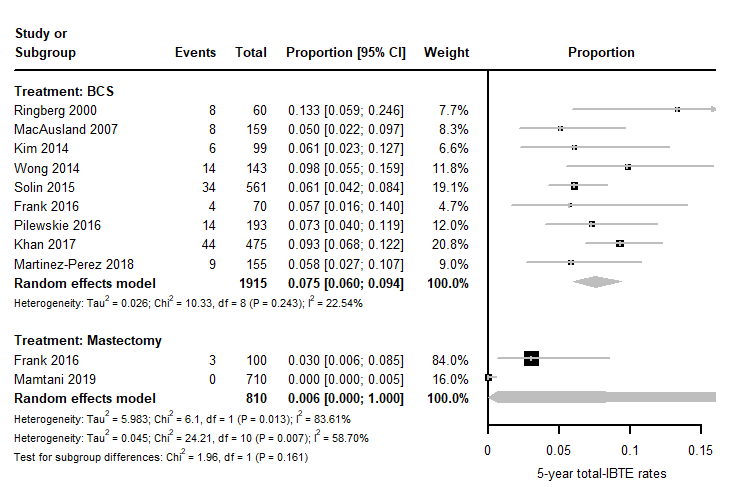


Supplementary Figure 10. Pooled analysis of 5-year total-IBTE rates in low-risk DCIS comparing BCS and mastectomy


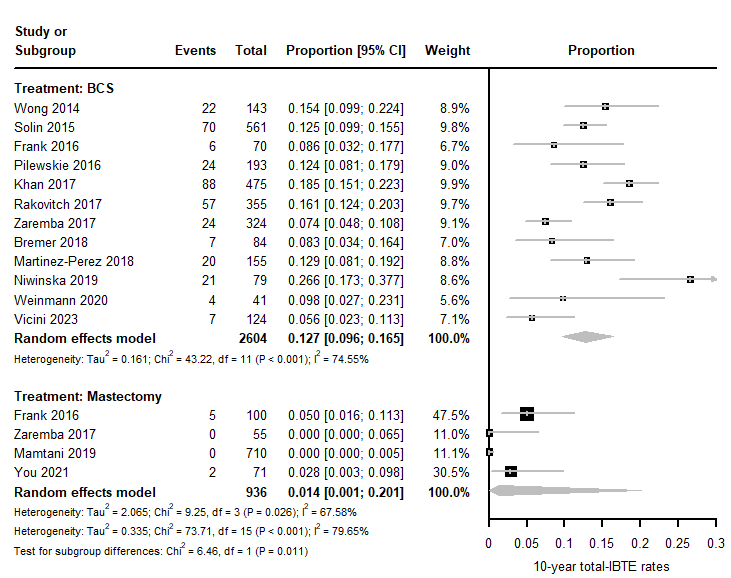


Supplementary Figure 11. Pooled analysis of 10-year total-IBTE rates in low-risk DCIS comparing BCS and mastectomy


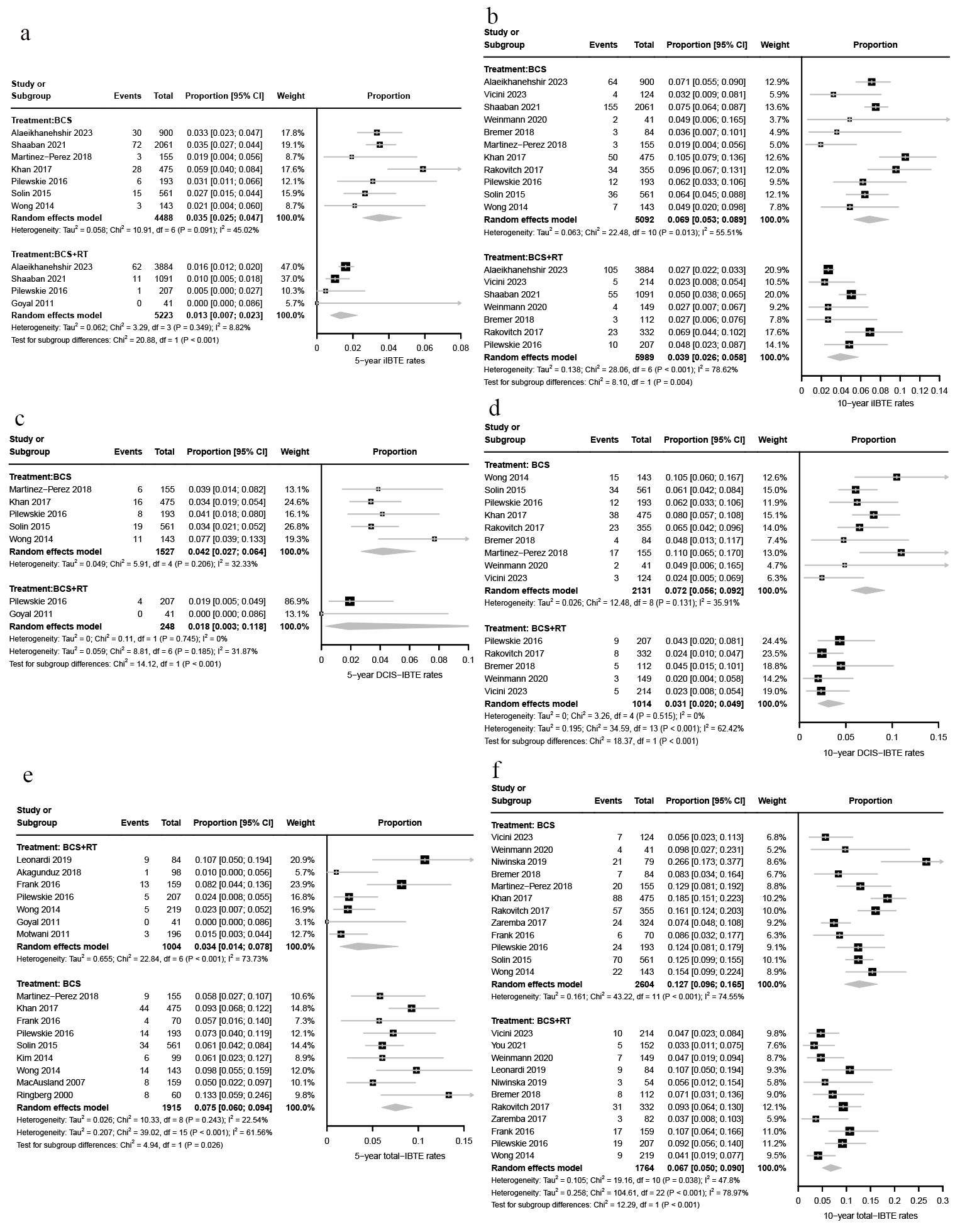


Supplementary Figure 12. Pooled analysis of 5-year and 10-year iIBTE rates (a, b), DCIS-IBTE (c, d) and total-IBTE (e, f) in low-risk DCIS comparing BCS and BCS followed by RT.


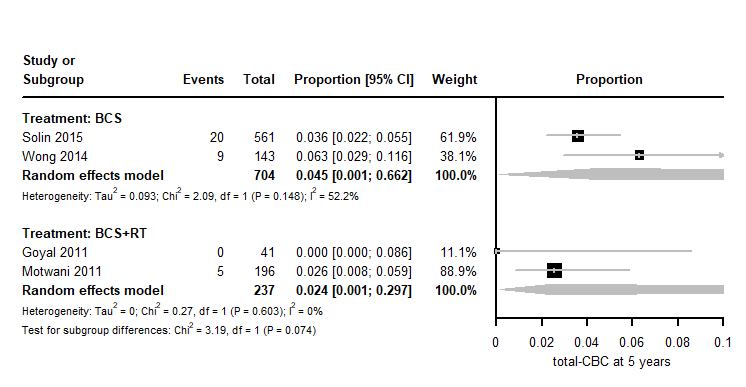


Supplementary Figure 13. Pooled analysis of 5-year total-CBC rates in low-risk DCIS comparing BCS and BCS followed by RT


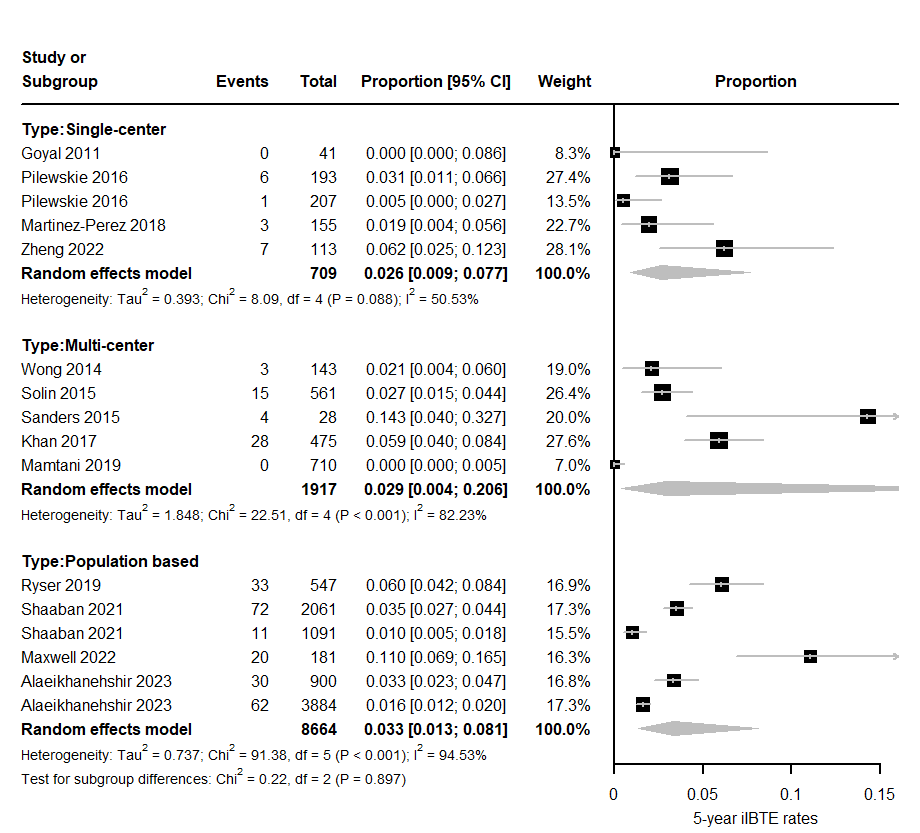


Supplementary Figure 14. Pooled analysis of 5-year iIBTE rates by study types


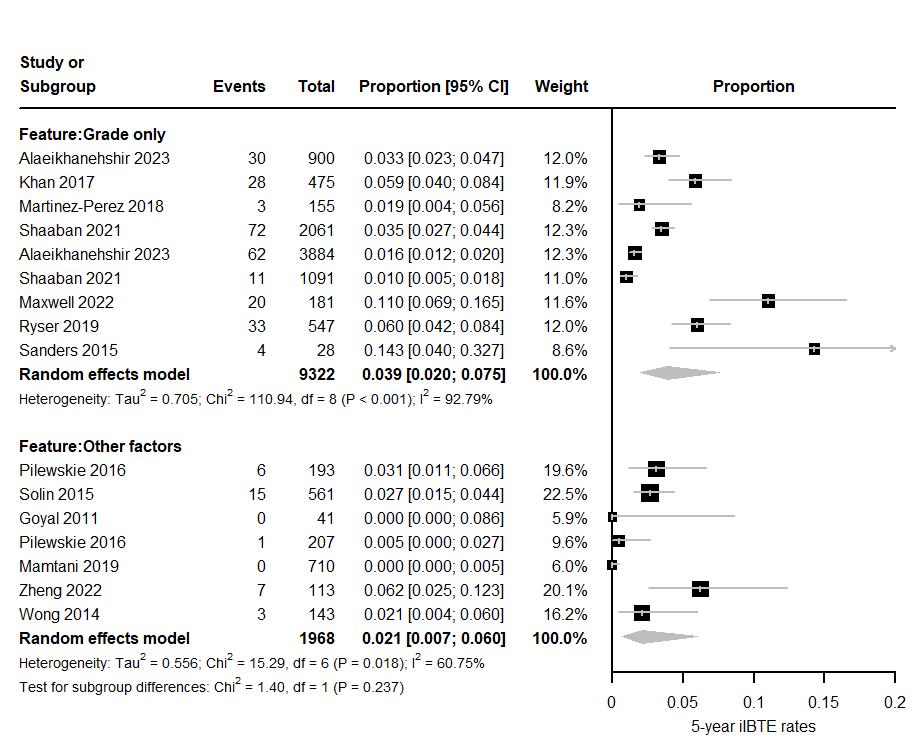


Supplementary Figure 15. Pooled analysis of 5-year iIBTE rates by the definition of low-risk


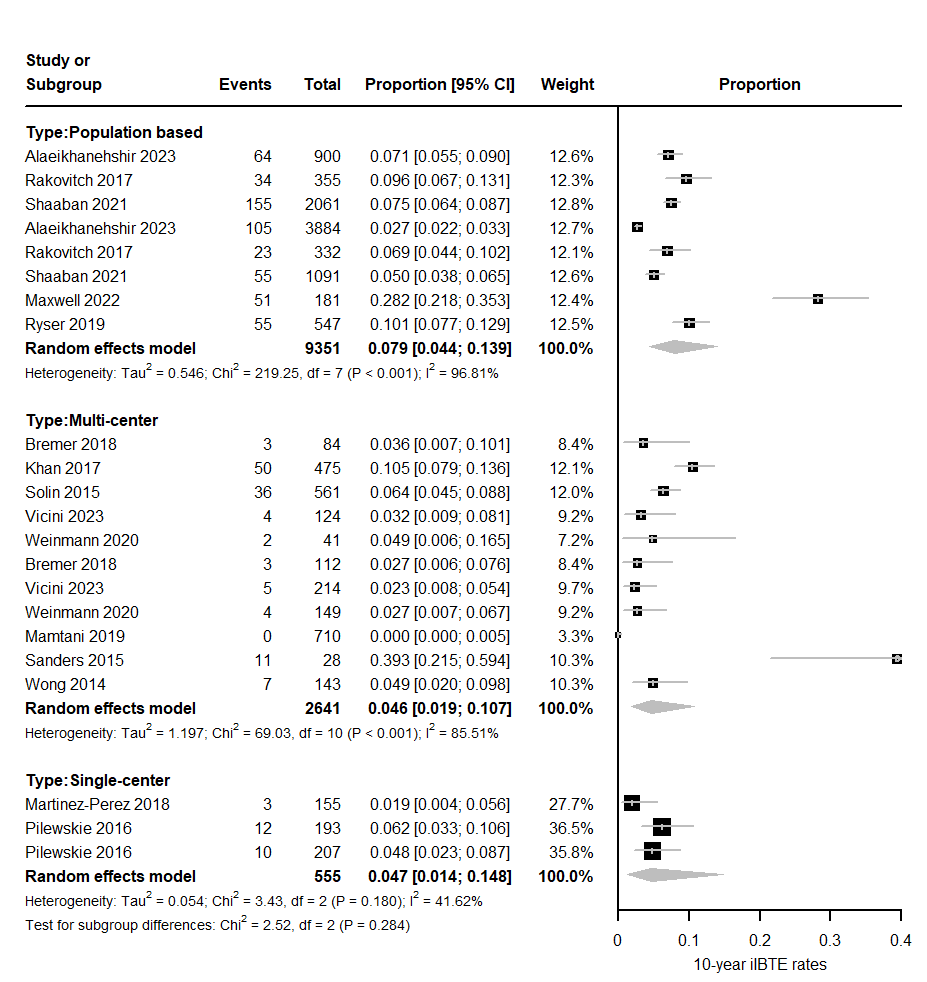


Supplementary Figure 16. Pooled analysis of 10-year iIBTE rates by study types


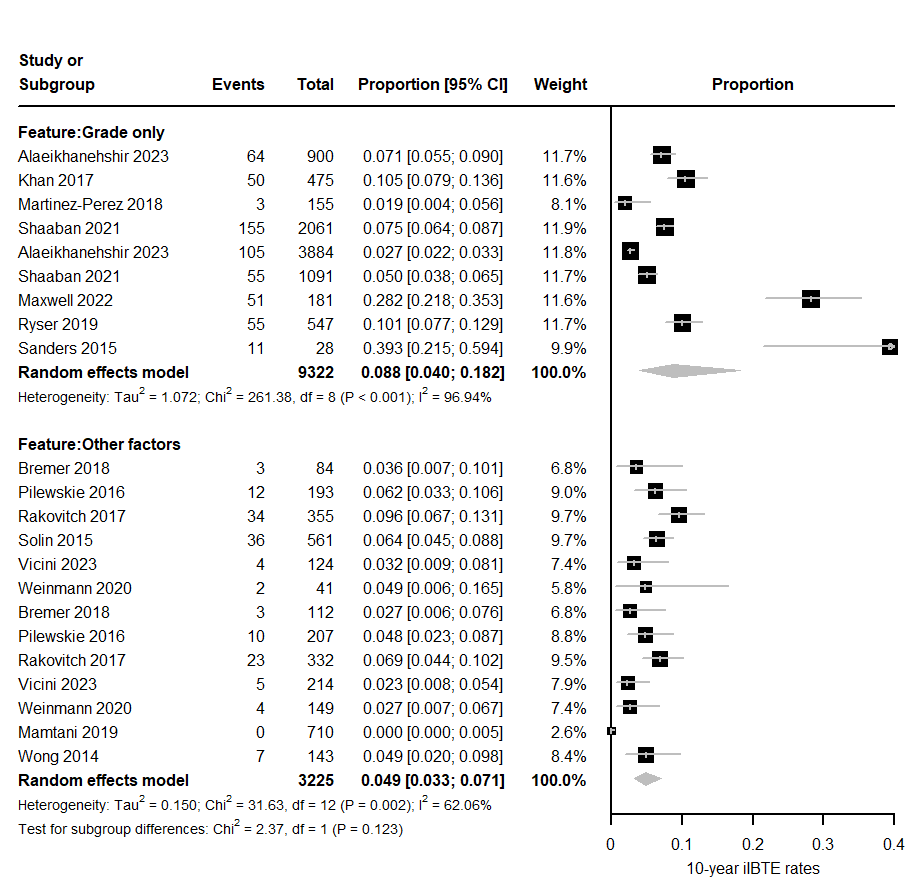


Supplementary Figure 17. Pooled analysis of 10-year iIBTE rates by the definition of low-risk


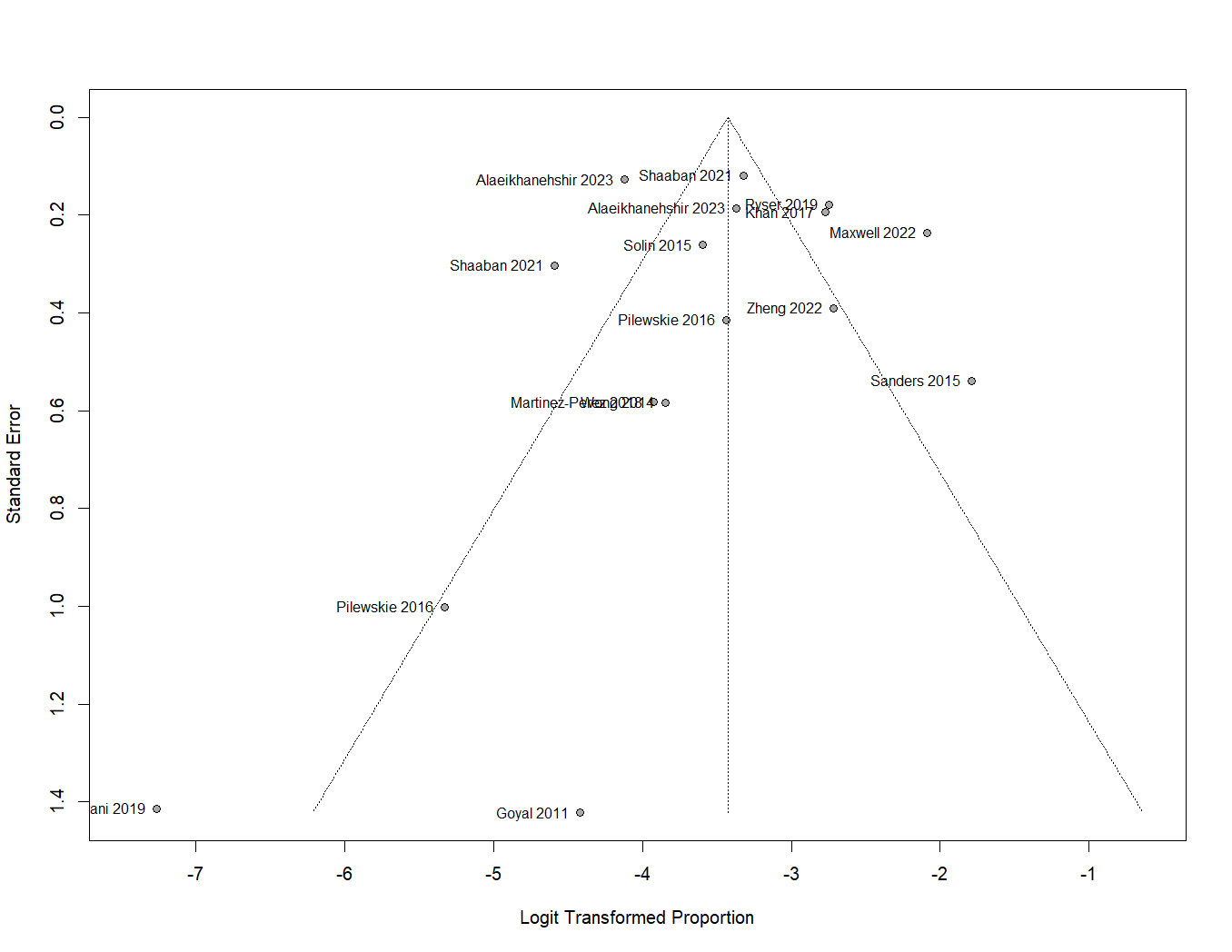


Supplementary Figure 18. Funnel plot of standard error by logit transformed of pooled 5-year iIBTE rate, t value of Egger’s test = -0.20, P = 0.8441


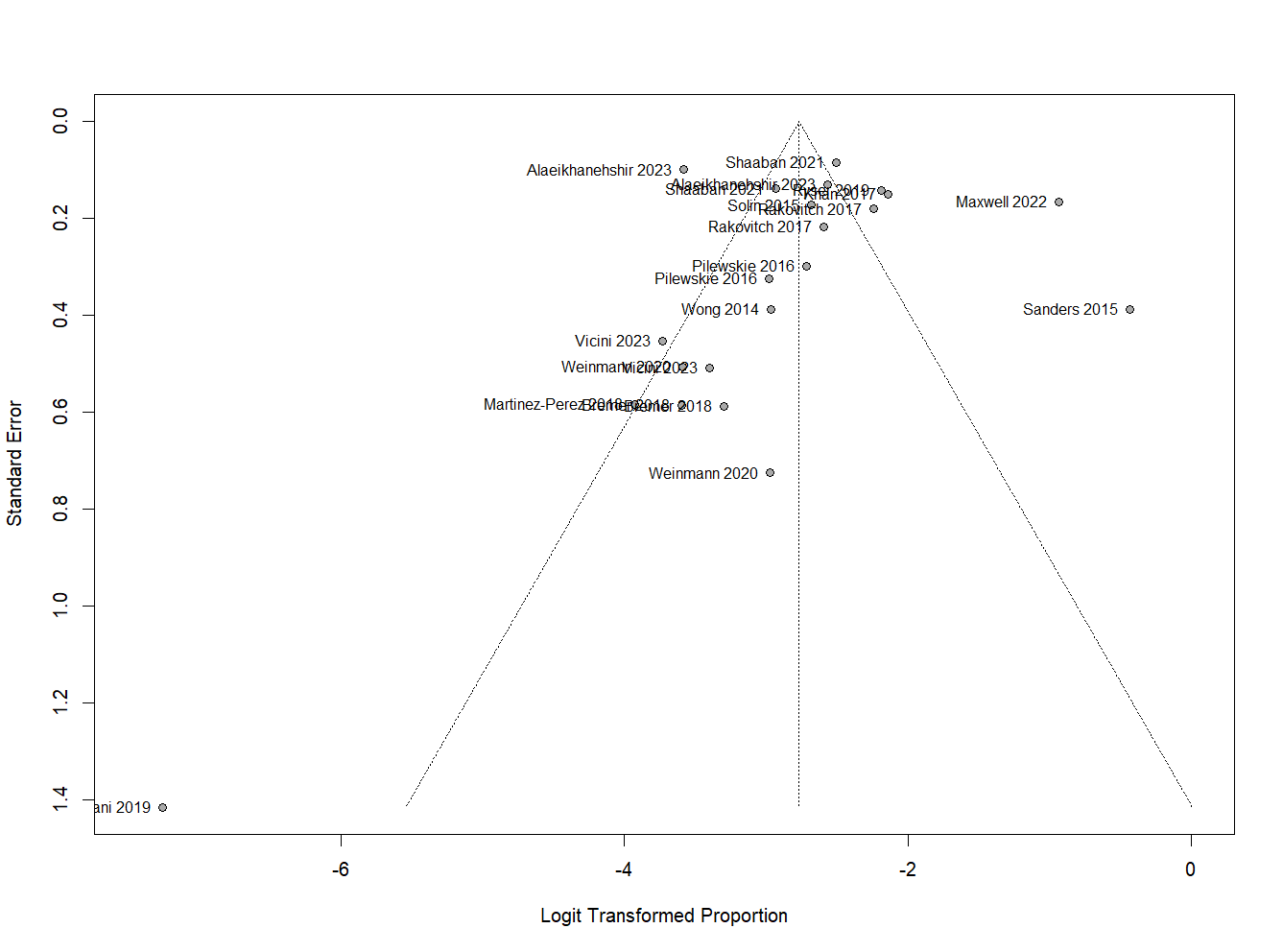


Supplementary Figure 19. Funnel plot of standard error by logit transformed of pooled 10-year iIBTE rate, t value of Egger’s test = -0.38, P = 0.7094


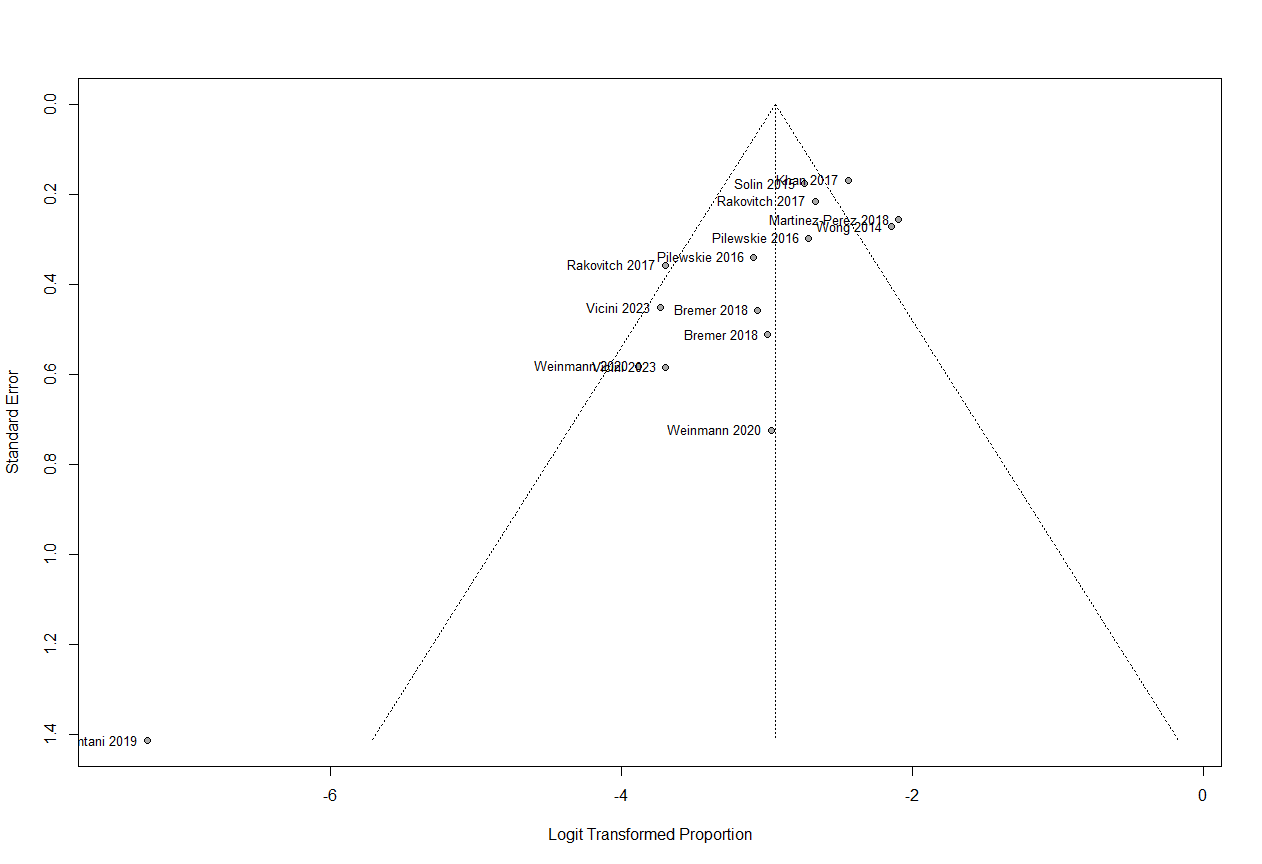


Supplementary Figure 20. Funnel plot of standard error by logit transformed of pooled 10-year DCIS-IBTE rate, t value of Egger’s test = -3.63, P = 0.0031


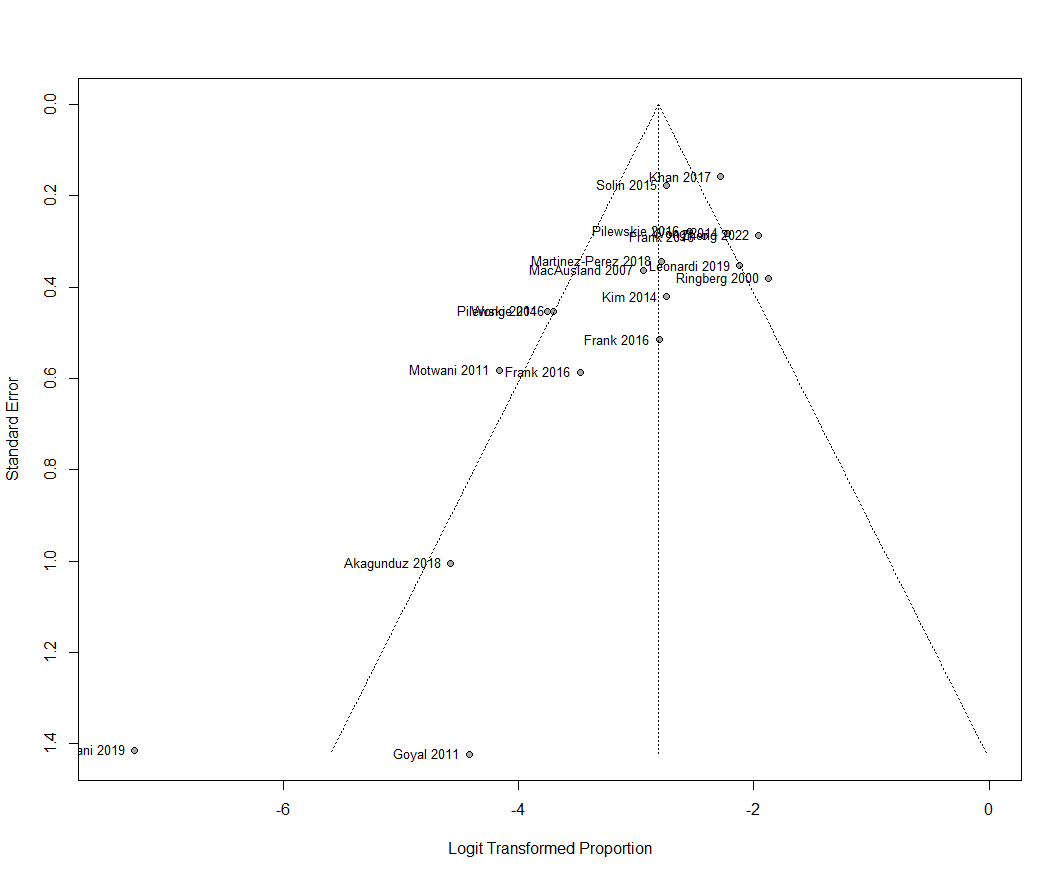


Supplementary Figure 21. Funnel plot of standard error by logit transformed of pooled 5-year total-IBTE rate, t value of Egger’s test = -3.59, P = 0.0023


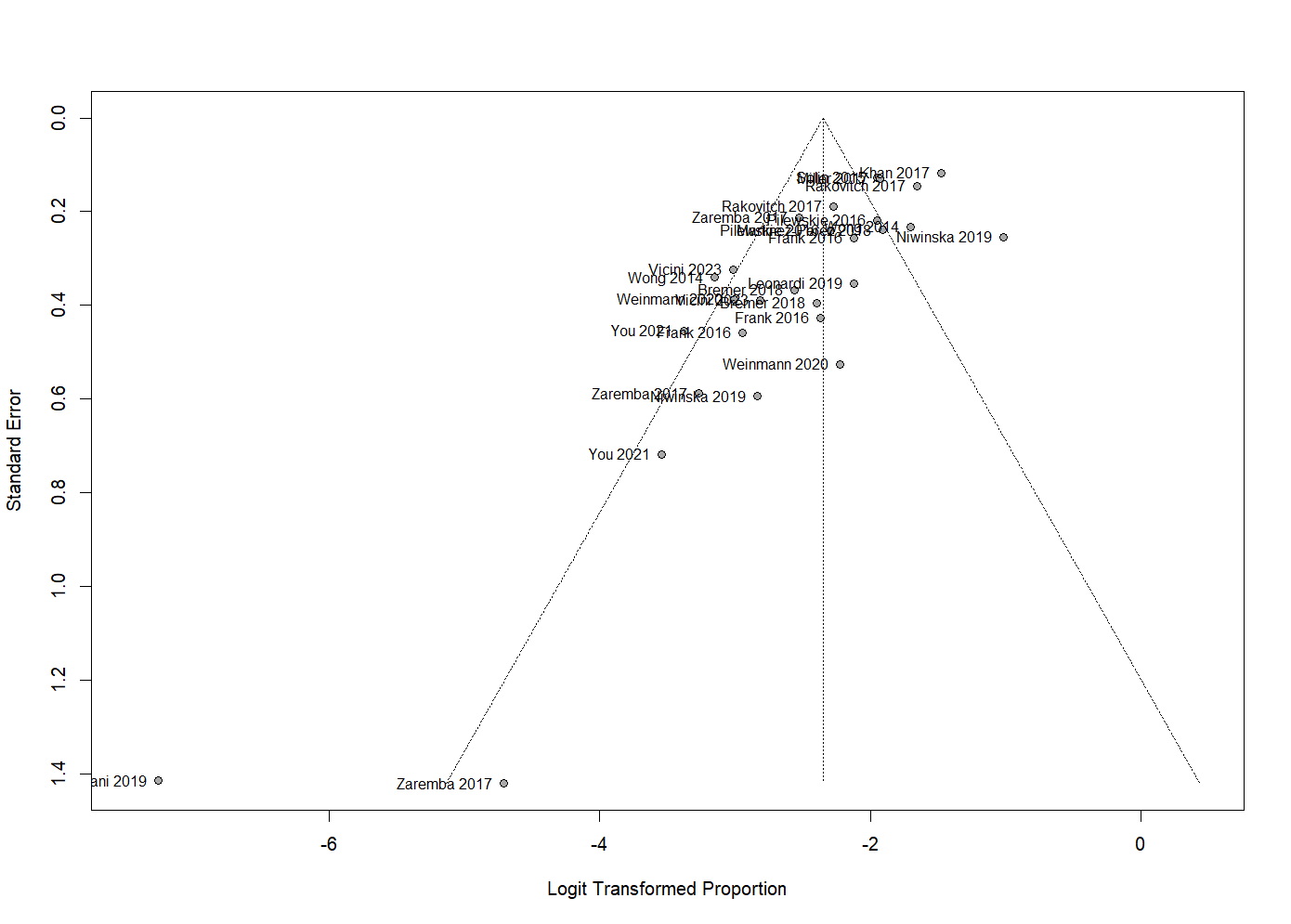


Supplementary Figure 22. Funnel plot of standard error by logit transformed of pooled 10-year total-IBTE rate, t value of Egger’s test = -5.53, P <0.0001.


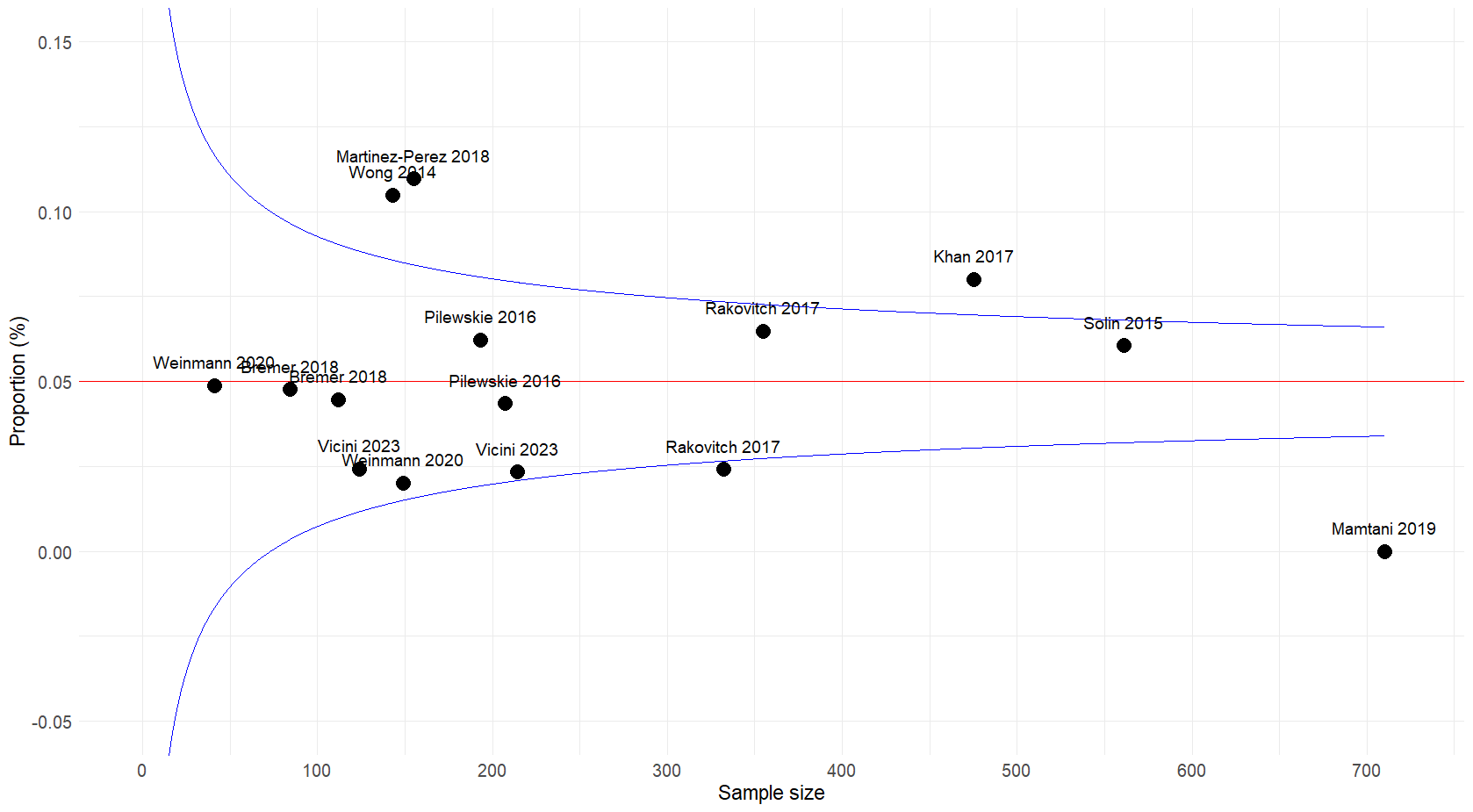


Supplementary Figure 23. Funnel plot of proportion by sample size of pooled 10-year DCIS-IBTE rates


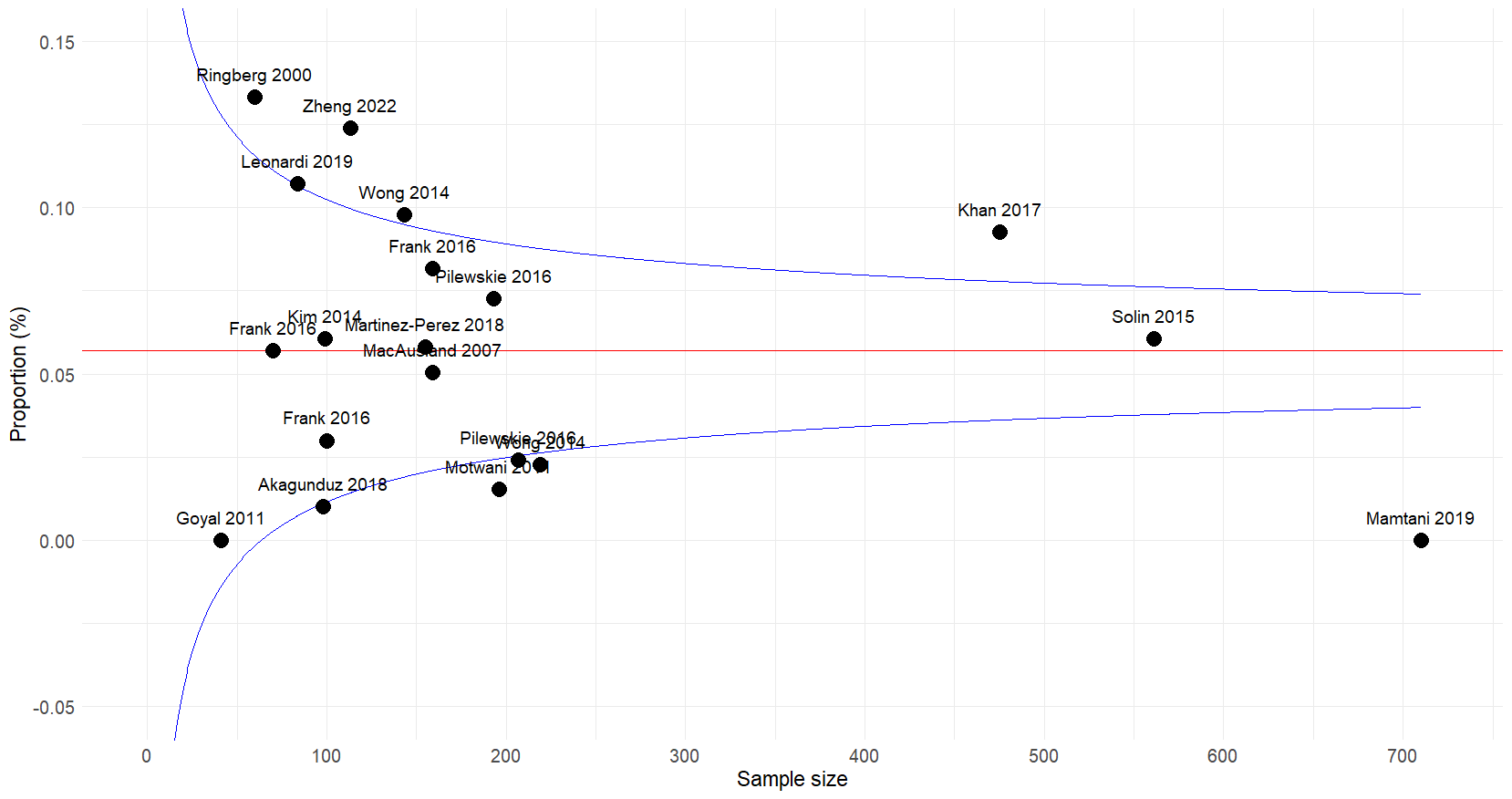


Supplementary Figure 24. Funnel plot of proportion by sample size of pooled 5-year total-IBTE rates


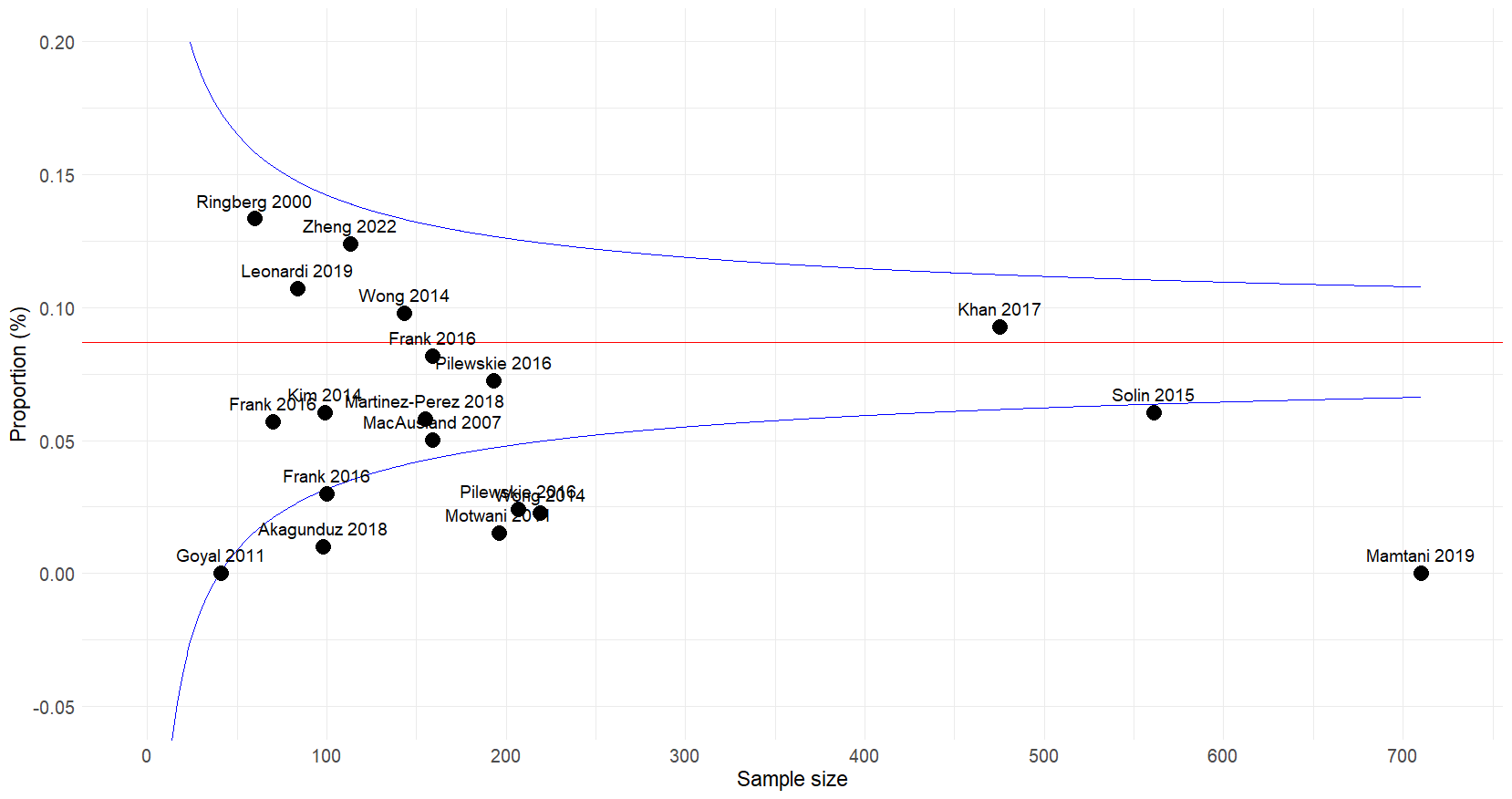


Supplementary Figure 25. Funnel plot of proportion by sample size of pooled 10-year total-IBTE rates


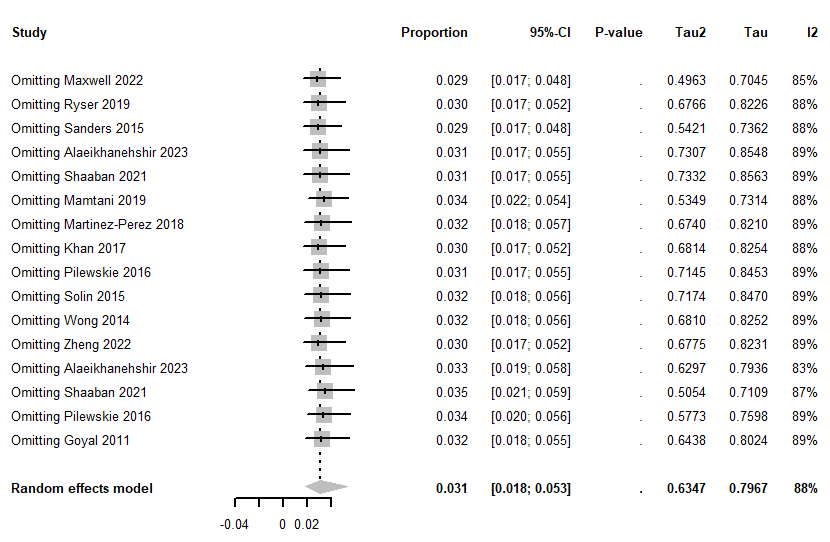


Supplementary Figure 26. Leave-one-out analysis of pooled 5-year iIBTE rates


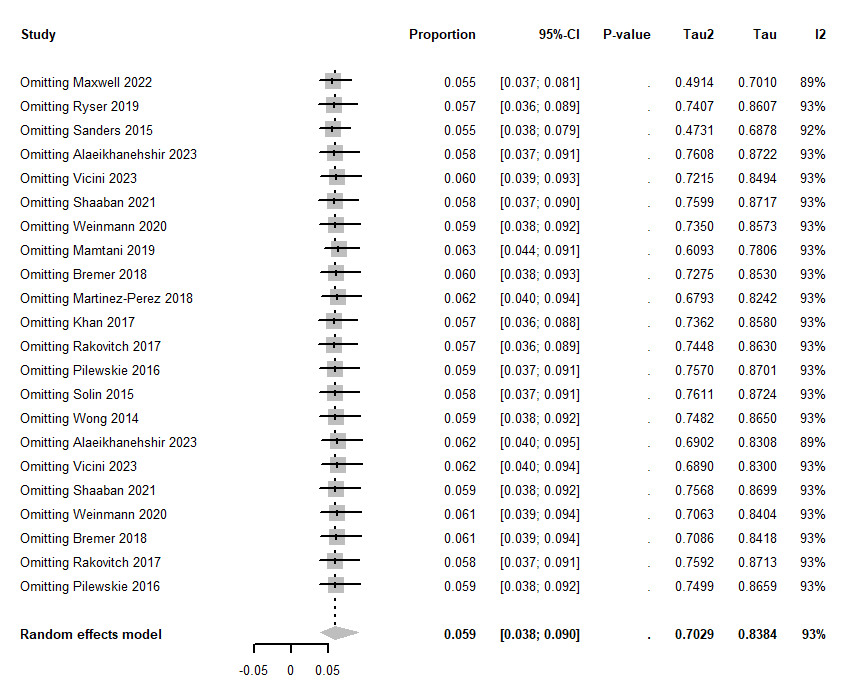


Supplementary Figure 27. Leave-one-out analysis of pooled 10-year iIBTE rates


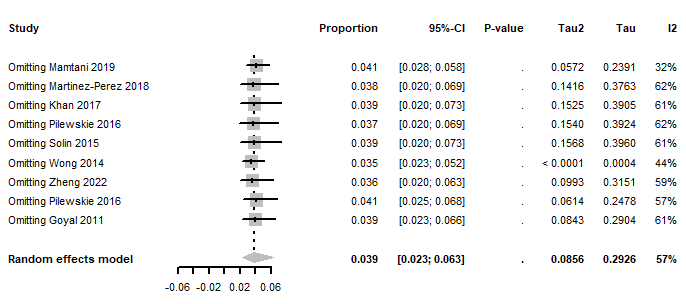


Supplementary Figure 28. Leave-one-out analysis of pooled 5-year DCIS-IBTE rates


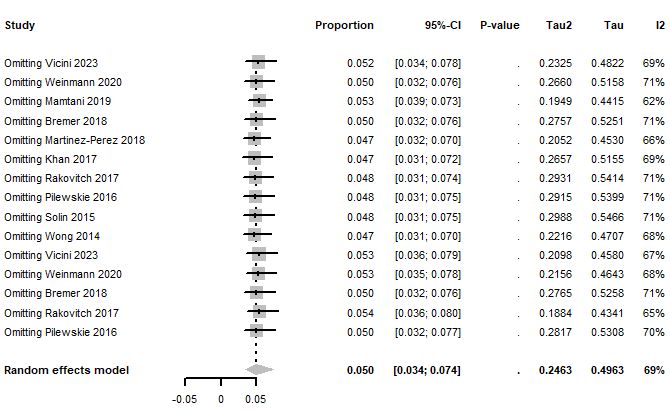


Supplementary Figure 29. Leave-one-out analysis of pooled 10-year DCIS-IBTE rates


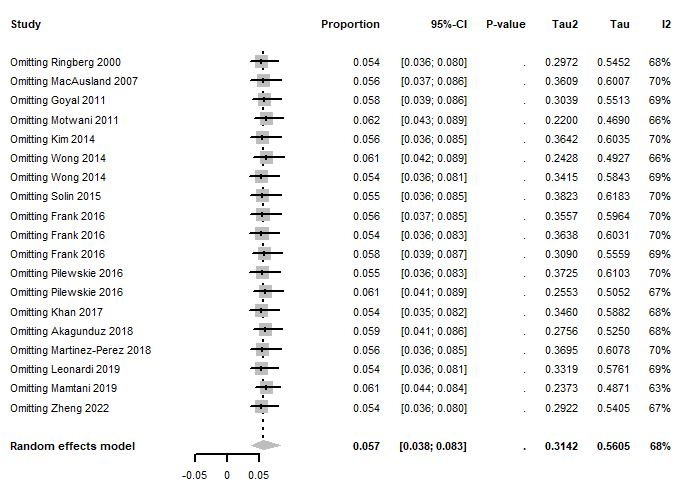


Supplementary Figure 30. Leave-one-out analysis of pooled 5-year total-IBTE rates


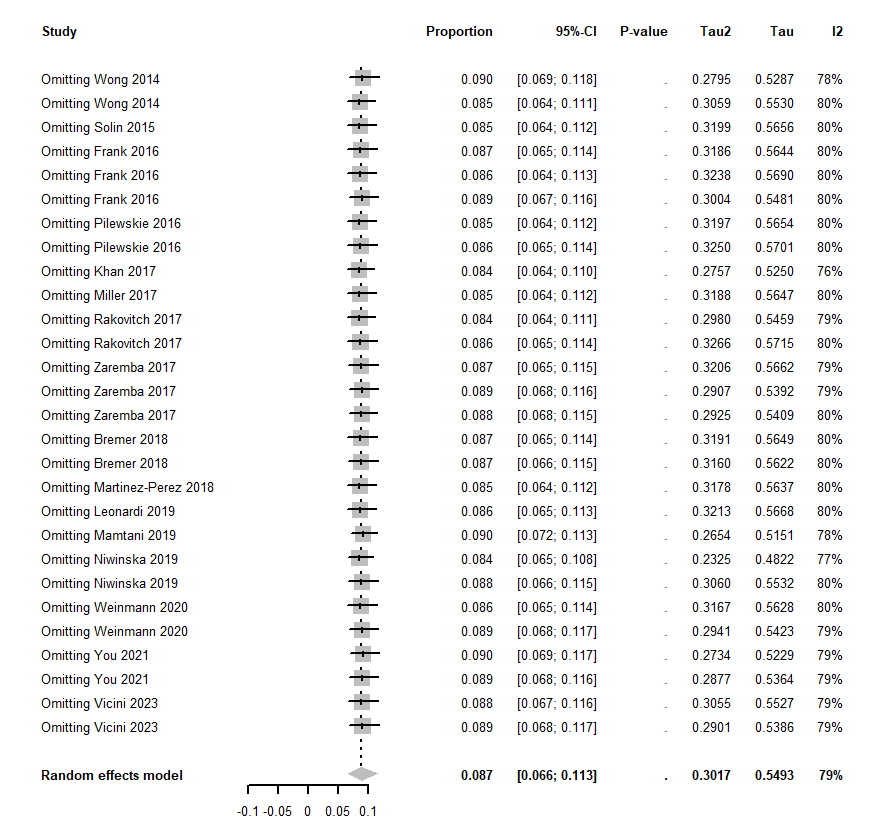


Supplementary Figure 31. Leave-one-out analysis of pooled 10-year total-IBTE rates


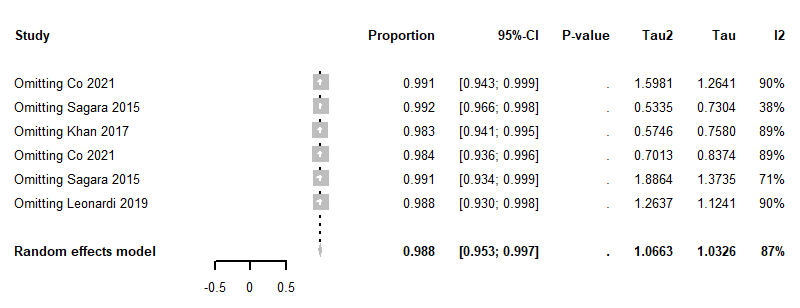


Supplementary Figure 32. Leave-one-out analysis of pooled 10-year BCSS rates
